# Lipid bilayer environments control exchange kinetics of deep cavitand hosts and enhance disfavored guest conformations†
†Electronic supplementary information (ESI) available: Spectral data not included in the text, detailed description of the exchange fitting process. See DOI: 10.1039/c7sc05155g


**DOI:** 10.1039/c7sc05155g

**Published:** 2018-01-11

**Authors:** Lizeth Perez, Bethany G. Caulkins, Magi Mettry, Leonard J. Mueller, Richard J. Hooley

**Affiliations:** a Department of Chemistry , University of California – Riverside , Riverside , CA 92521 , USA . Email: richard.hooley@ucr.edu

## Abstract

The effects on the molecular recognition properties of water-soluble deep cavitand hosts upon embedding them in phosphocholine lipid bilayer environments have been studied by 2D NMR experiments. By employing suitable guests containing ^19^F or ^13^C nuclei that can be encapsulated inside the host, 2D EXSY NMR experiments can be used to analyze and compare the in/out guest exchange rates in aqueous solution, isotropically tumbling micelles, or magnetically ordered bicelles. These analyses show that embedding the deep cavitands in lipid bilayers slows the guest exchange rate, due to the lipids acting as a “compression sleeve” around the host, restricting guest egress. This effect also enhances guest conformations in the host that are not observed in free solution, such as axial cyclohexane conformers and ketone hydrates.

## Abstract 2

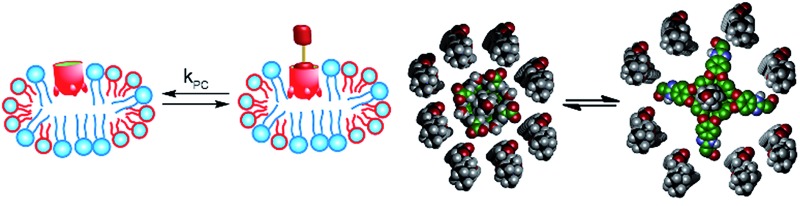
External lipid membranes affect guest recognition properties of water-soluble hosts, enhancing molecular conformations and equilibria not observed in free solution.

## Introduction

When analyzing the conformation and motion of molecules confined in small spaces, the predominant tool is NMR spectroscopy.^1^ The sensitivity of ^1^H NMR experiments, along with the large changes in proton chemical shifts possible upon surrounding small hydrocarbons with aromatic π clouds, have opened a window into the physical behavior of molecules in enclosed environments. Quantitation of the thermodynamics^2^ and kinetics2a,^3^ of substrate binding is possible, as well as investigations into the orientation,^4^ conformation,^5^ motion2a,^6^ and unusual isomerism^7^ of bound small molecule substrates. Molecular confinement can lengthen the lifetime of reactive intermediates^8^ and unstable species:^9^ observing these phenomena often relies on ^1^H NMR spectroscopy. To maximize detection, these investigations are generally performed in controlled environments, in deuterated solvents and in the absence of NMR-visible additives and impurities.

**Fig. 1:**
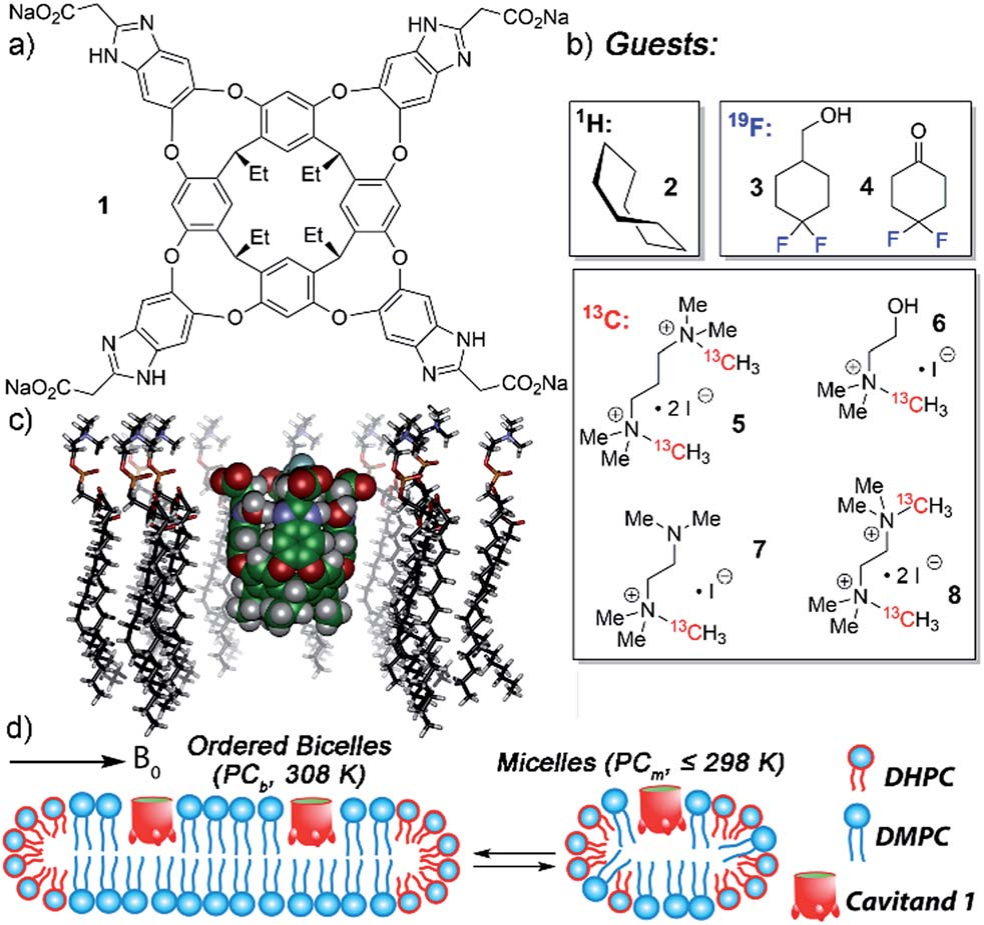
Structure of (a) water-soluble deep cavitand **1**; (b) guests used in this study; (c) representation of **1** embedded in a DMPC lipid monolayer (SPARTAN, AMBER forcefield); (d) representation of possible DMPC/DHPC lipid structures, either magnetically ordered bicelles or isotropically disordered micelles.

Synthetic host molecules are capable of selective molecular recognition in far more complex and challenging environments than pure solvent, however. Hosts such as deep cavitand **1** (Fig. 1)2a,^10^ have been shown to bind targets while embedded in supported phosphocholine (PC) lipid bilayers^11^ and even in living cells.^12^ The recognition capabilities of cavitand **1** in membrane bilayer systems have been shown *via* indirect methods, such as surface plasmon resonance (SPR) spectroscopy of cavitand:supported lipid bilayer (SLB) aggregates^11^ and capillary electrophoresis (CE) of liposome:cavitand systems.11*d* Other techniques can also be used, including fluorescence spectroscopy^13^ and isothermal calorimetry.2c,10a While these techniques have their advantages, none of them are as enticing as NMR spectroscopy, which allows sensitive interrogations of guest conformations, motions and dynamics, such as in/out exchange rates and the molecular motion of molecules bound in the host's interior. ^1^H NMR spectroscopy of these events has been limited to simple 1D experiments in fast tumbling micelles,^14^ although some examples of molecular recognition in more complex systems such as human serum or urine^15^ are known.

The tetracarboxylate cavitand **1** is soluble in water up to ∼20 mM, but is highly lipophilic, and is smoothly incorporated into a variety of lipid aggregates.^11^,^12^ It can bind a wide range of suitably sized guest molecules in aqueous solution, ranging from hydrocarbons2*a* to substituted trimethylammonium (R-NMe_3_^+^) salts such as choline.^10^,^12^ In pure D_2_O solution, the association constants of guests such as 1-adamantanemethanol (*K*_a_ = 2.9 × 10^5^ M^–1^), cyclohexanone (*K*_a_ = 1.6 × 10^5^ M^–1^), choline (*K*_a_ = 2.6 × 10^4^ M^–1^) and acetylcholine (*K*_a_ = 1.2 × 10^5^ M^–1^) are relatively consistent, within an order of magnitude or so.2a,10b The “upper limit” for guest association in free solution is on the order of 2 × 10^5^ M^–1^. The in/out kinetics of bound guests, as well as their motion while inside the cavity, have been extensively investigated in pure D_2_O by 2D NMR techniques.2a,4a The mechanism of in/out exchange is a dissociative process, independent of guest concentration.2*a* The cavitand releases guest *via* an “S_N_1-like” mechanism, whereby the walls flex open to unfold the cavitand in the rate-determining step, followed by rapid guest exchange. In water, the energy barrier consists of three major components: the energy barrier to rotate around the C–O bonds in the cavitand walls^16^ (∼11 kcal mol^–1^), plus the energetic penalties of unfavorable solvation of the cavitand walls and the bound guest once unfolded. Depending on guest size, these barriers range from 16.0–17.2 kcal mol^–1^,2a,^17^ with observed rates ranging from 1.8 s^–1^ (adamantanol) to 14.6 s^–1^ (cyclohexane). All these investigations used ^1^H NMR for analysis, as the ^1^H peaks for bound guest are shifted strongly upfield, and are easily visible at negative ppm, unhindered by ^1^H peaks from the host or excess free guest.

What is not known is how embedding the cavitand in a lipid bilayer affects these molecular recognition properties. Other techniques have shown that host is capable of guest recognition in a bilayer, and that guest binding affinities are enhanced in some cases,^11^ but the effect on guest in/out kinetics and conformation is unknown. Cavitands are excellent mimics of proteins, and have shed light on many biomimetic recognition phenomena. Can they be used to illustrate the function of membrane-binding proteins, a far more elusive target?

NMR analysis of (bio)molecular structure and dynamics in membrane bilayers is well-studied,^18^ and exploits such biomimetic environments as isotropically tumbling^19^ and magnetically-oriented lipid bicelles, and unaligned and mechanically oriented phospholipid bilayers.18a,^20^ The aligned systems have the advantage of high resolution without the requirement of either fast isotropic reorientation (which restricts the dimensions of isotropic bicelles) or magic angle spinning (used for unaligned bilayers). These experiments often require isotopically enriched species for detection, and the nuclei of choice are generally ^13^C, ^15^N or ^31^P. Unfortunately, observing individual proton signals *via*^1^H NMR analysis of the binding processes in those cases is complicated by the presence of multiple hydrocarbon peaks from the lipids. More sensitive NMR experiments are rendered impractical by line broadening, and so dynamic NMR experiments that are essential for analysis of guest motion and binding kinetics in synthetic cavity-containing hosts are challenging. Here we employ a variety of guest molecules with different detectable nuclei for molecular recognition in a deep, water-soluble host, and investigate the effect of embedding the host in biomimetic membrane environments on the guest dynamics, conformation and reactivity.

## Results and discussion

NMR analysis of the molecular recognition of small molecules by deep cavitands in lipid environments is complicated by a number of factors. The guest must bind in the cavitand, and be detectable when bound inside the host in multiple different lipid environments. In addition, study of the in/out kinetics requires guests that are sufficiently soluble to display peaks for both free and bound guest. Study of internal motion or conformational bias requires guests that display multiple conformations or orientations while bound inside the cavity: if ^1^H NMR analysis cannot be used, this introduces serious constraints on the nature of the guest. As such, we investigated a wide range of guest species (Fig. 1) for their suitability. The guest library consists of simple hydrocarbons such as cyclooctane **2**, ^19^F-containing hydrophobic guests **3-4**, and ^13^C-enriched R-NMe_3_^+^ guests **5–8**. Each of these guests are either commercially available or accessible in one or two steps from commercial materials (see Experimental section for synthesis and characterization). Also, the host:guest library must be paired with a suitable membrane environment for analysis. Obviously, a natural cell membrane is challenging to use, but a number of surrogates are known and used for NMR analysis of membrane-bound biomolecules. One of the most effective mimics is a magnetically oriented bicelle, which maintains the bilayer sheet form of natural membranes while aligning in the magnetic field to allow analysis by ssNMR techniques.^20^ In addition, solution-phase NMR techniques can be employed with isotropically tumbling micelles. Each aggregate has benefits and drawbacks: bicelles are better mimics of natural membranes, but suffer from solid-state line broadening effects, whereas micelles are easier to analyze but are an imperfect membrane mimic. Fortunately, both bicelles and micelles can be accessed from the same lipid system, so we applied both types of lipid environment to the host:guest analysis.

The lipid aggregates were formed from a 3.2 : 1 mix of dimyristoylphosphocholine and diheptylphosphocholine lipids (DMPC/DHPC), which are well-known to allow formation of both magnetically oriented bicelles and smaller disoriented micelles.^19^ As illustrated in Fig. 1d, the type of lipid aggregate formed is dependent on temperature, with magnetically ordered bicelles dominant at 308 K, and disordered (isotropic) micelles favored at 298 K or lower. This can be seen by ^31^P NMR analysis of the lipid system. The disordered micelles display only one averaged ^31^P phosphate peak (see ESI, Fig. S-47 and S-48†), whereas the oriented bicelles display two peaks, due to the two different orientations of phosphate groups in the bicelle. The assembly and structure of these aggregates was not visibly affected by the presence of either 5 mM cavitand **1** or 5 mM **1** + 7 mM guest **6**.

^31^P NMR analysis of the lipid phosphate groups is an invaluable tool to confirm the structure of the lipid aggregates, but does not allow investigation of the host:guest properties of **1**. We initially approached the host:guest studies using ^1^H NMR spectroscopy with the simplest, most optimal guest possible. Cyclooctane **2** is easily extracted into the cavity of **1**, has an affinity >10^4^ M^–1^ by NMR,2*a* and tumbles rapidly on the NMR timescale, showing a single bound peak at –1.50 ppm corresponding to the averaged signal of all 16H in the guest. A premade sample of **1·2** in D_2_O was added to solutions of either DMPC:DHPC micelles (hereinafter denoted as PC_m_) or DMPC:DHPC bicelles (PC_b_) for a final [**1**] = [**2**] = 1.8 mM and the spectra acquired at 283 K and 308 K respectively (see ESI† for spectra). The spectrum in PC_m_ shows that cavitand **1** binds **2** strongly in the hydrophobic lipid environment, as the expected sharp singlet for the **1·2** is retained. The cavitand is completely incorporated into the aggregates under the conditions used. T2-filtered spectra of the **PC_m_·1·2** complex (see Fig. S-26†) show no peaks for either free, un-embedded cavitand or bound guest, indicating that all the detectable host is incorporated into the lipid aggregates under the conditions used. Unfortunately, no change in the conformational properties of **2** was observed: even at 10 °C, the guest tumbled rapidly in the cavity of **1**. In addition, in the bicellar environment at 35 °C, only broad undefined peaks could be observed and no discrete peaks for **1** or bound **2** are visible, even when magic angle spinning (MAS) was applied.

As cyclooctane **2** was only partially useful, we turned to guests **3** and **4**, containing ^19^F nuclei. As ^19^F peaks are broadened in the solid state in a similar manner to ^1^H, we focused on fast-tumbling micelles in the **1·3**/**4** analysis, with a lower lipid concentration of 60 mg mL^–1^ for ease of measurement. To allow analysis of conformation and in/out guest exchange, the guest must contain ^19^F nuclei that are bound inside the cavity of **1**, and display the characteristic chemical shift variations caused by the magnetic anisotropy of the host. Fortunately, both guests **3** and **4** are suitable guests for **1** in aqueous solution, albeit displaying weaker binding than the equivalent hydrocarbons. ^19^F NMR spectra of the host:guest complexes shows that the bound ^19^F nuclei are upfield shifted, Δ*δ* ∼ –2 ppm, indicating that the difluorocyclohexanyl group is oriented to the cavity interior. Presumably, the OH and C=O groups orient towards the external solvent to benefit from favorable H-bonding.

**Fig. 2:**
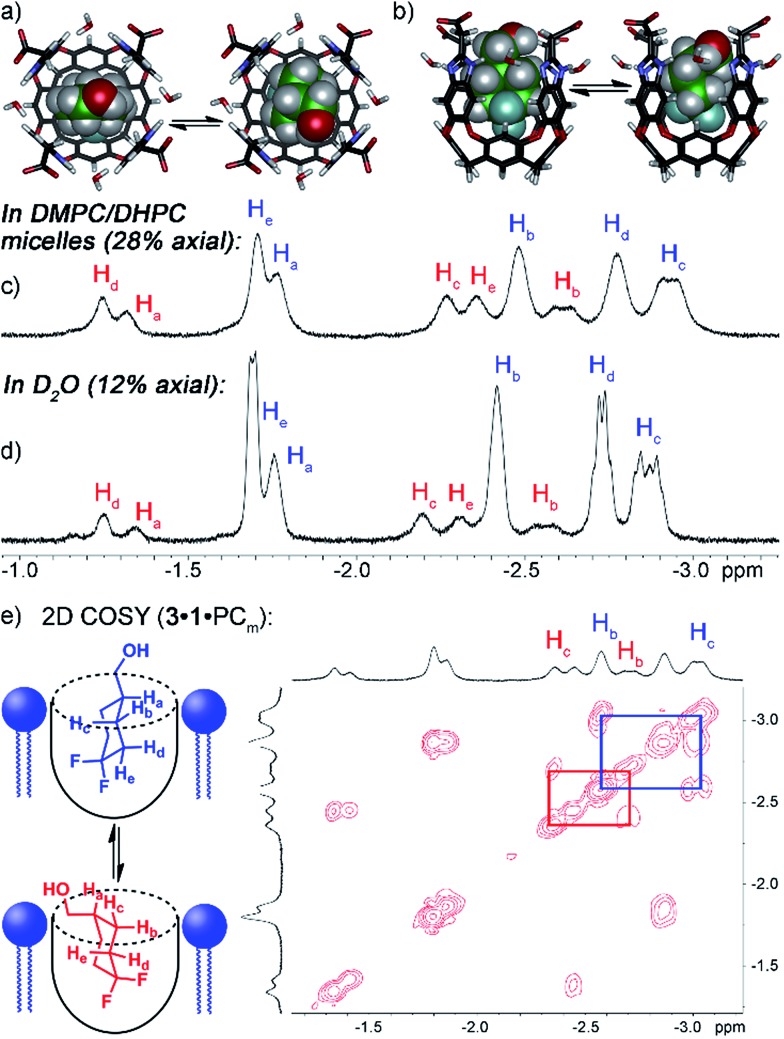
Enhanced axial conformation of bound guest **3**. (a and b) Minimized structures of the **1·3_eq_** and **1·3_ax_** complexes (SPARTAN, AMBER forcefield). Upfield regions of the ^1^H NMR spectra of (c) **PC_m_·1·3**; (d) **1·3**; (e) 2D COSY spectrum of **PC_m_·1·3** (700 MHz, 298 K, [**1**] = 5.8 mM, [**3**] = 39.5 mM, ratio DMPC/DHPC = 3.2 : 1, 60 mg mL^–1^ total lipid concentration).

Both guests **3** and **4** show interesting and unexpected behavior when bound to **1**, and provide excellent examples of the effect of embedding the host in the PC lipid environment. The ^1^H NMR spectrum of the **1·3** complex (Fig. 2d) showed two sets of peaks for bound **3**. The ^1^H chemical shifts for the guest CH peaks in each conformation were relatively similar, indicating that the two conformations in the host:guest complex are not up/down carceroisomers,4a,^21^ rather the axial/equatorial ring flip conformers of **3**. This observation was unexpected, and provided an opportunity to investigate the effects of molecular recognition on conformations of bound guest. In the absence of host in either CDCl_3_ or D_2_O solution, only one conformation of **3** can be observed in the ^1^H or ^19^F NMR spectrum, presumably that of the lower energy equatorial conformer. At the concentrations used, this indicates that <0.5% of the axial conformer is present in solution. The ^19^F spectrum is most useful for this assignment: the peak for the axial F is a doublet of triplets (see Fig. S-2†), due to *trans*-diaxial coupling with the vicinal H atoms. The equatorial F is a doublet, and shows no visible peak splitting due to coupling to the protons. The highly different coupling patterns shown by the two fluorines indicate that no appreciable rapid interconversion between conformers is occurring. In contrast, when bound in the cavity of **1**, 12% of the population of **1·3** corresponds to the axial conformer. 2D COSY analysis (Fig. 2e) clearly shows the two separate conformers, and molecular modeling (Fig. 2a) illustrates that the axial conformer of **3** easily fits inside the host cavity. The behavior of guest **3** in the **PC_m_·1** system gives the first indication of the effect of embedding the host in lipid environment. Fig. 2c shows the upfield region of the ^1^H spectrum of **PC_m_·1·3**, and it is immediately obvious that the proportion of axial conformer is greatly increased when compared to that seen in the **1·3** complex. In this case, 28% of the bound **3** exists in the axial conformer, compared to only 12% in **1·3** and <0.5% in free solution.

**Fig. 3:**
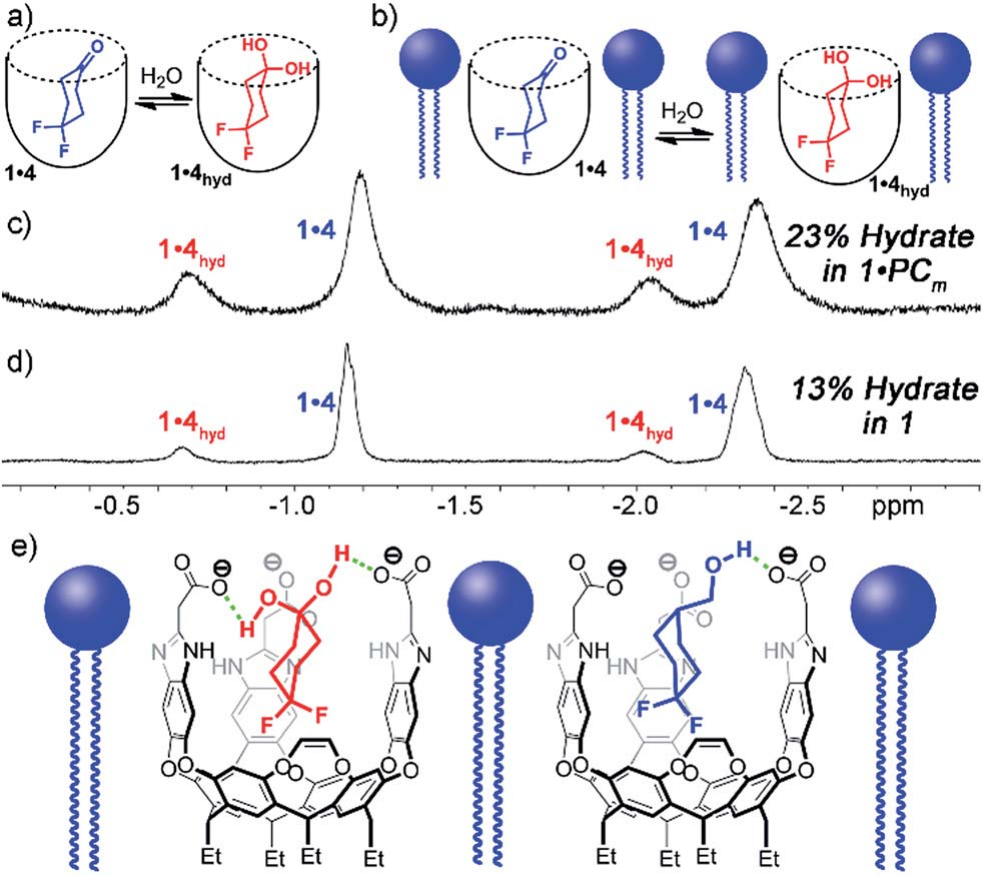
Enhanced hydration of bound guest **3**. (a and b) Illustration of the equilibrium process. Upfield regions of the ^1^H NMR spectra of (c) **PC_m_·1·4**; (d) **1·4** (400 MHz, 298 K, [**1**] = 5.8 mM, [**4**] = 39.5 mM, ratio DMPC/DHPC = 3.2 : 1, 60 mg mL^–1^ total lipid concentration) (e) illustration of the favorable hydrogen bonding present in **PC_m_·1·4_hyd_** and **PC_m_·1·3_ax_**.

Interestingly, the ^1^H and ^19^F NMR spectra of the **1·4** complex are reminiscent of those of **1·3**, in that two different guests are bound, even though only one was seen in free solution. Again, the chemical shifts of the CH protons in the bound guests do not match the expected signals for up/down carceroisomers, but represent the recognition of **1·4** and **1·4_hyd_**, namely the ketone and hydrated *gem*-diol form (Fig. 3). The ^19^F spectrum of hydrated **1·4_hyd_** is highly reminiscent of **1·3**: whereas the ^19^F peaks for cyclohexanone **1·4** are close in shift, the all-sp^3^ cyclohexane skeleton of **1·4_hyd_** (similar to that of **1·3**) separates the two fluorine peaks to reflect the distinct axial and equatorial positions (see Fig. 5 for full spectrum). The equilibrium between these two states strongly favors the ketone form in the case of unactivated ketones such as acetone, but the presence of the electron-withdrawing groups such as halogens increases the favorability of the hydrate (*K*_hyd_(acetone) = 1.4 × 10^–3^, *K*_hyd_(fluoroacetone) = 0.11).^22^ NMR analysis of guest **4** in D_2_O in the absence of cavitand showed no obvious hydrate present. At the concentrations used, this indicates that <0.5% hydrate is present in solution. In contrast, when bound inside the cavity of **1**, 13% of bound **4** exists in the hydrated form at 298 K. Embedding the host in PC lipids also biases the hydration equilibrium of **4**, similar to the conformational bias seen in the binding of **3**. When **4** was added to the **1·**PC_m_ system, 23% of the bound **4** was present in the hydrated form, compared to only 13% in **1·4** and <0.5% in free solution.

These observations introduce the question of why binding in **1** stabilizes normally unfavorable guest structures, and why this effect is enhanced when **1** is embedded in lipid aggregates. Cavitand **1** is well-known to display dual-mode recognition, whereby both the defined cavity and the upper rim groups can affect guest binding.13*a* The upper rim carboxylates have been shown to accelerate solvolysis reactions of bound guests,4*a* and control binding selectivity for functionalized guests in different pH conditions.13*b* The presence of anionic carboxylate functions at the upper rim of the cavity confers favorable H-bonding to acidic groups in the guest positioned in close proximity, more so than the external bulk water. Guests containing properly positioned H-bond donors such as ammonium ions,10b,13a thioureas^13^ or even hydroxy groups2a,4a,10b,13a have been shown to have stronger affinity for **1** than those with esters, ethers or ketones.4a,10b,13a The axial conformer of **3** evidently positions the OH group in closer proximity to the rim carboxylates, increasing the favorability of that conformer when bound. The hydrated *gem*-diol of **4** is capable of H-bonding with the carboxylates, whereas the ketone is not, hence the increased favorability of the bound hydrate (Fig. 3e).

Why this effect is enhanced when **1** is bound inside lipid micelles is less clear, but two possibilities present themselves. The cavitand could be positioned in the bilayer such that a small “hydrophobic” pocket is created above the cavitand rim, hiding the bound guest somewhat from external water and increasing the effect of the H-bonding between guest and host by limiting competitive H-bonding with the external water. This theory was previously used to explain the enhanced binding of cationic proteins to a **1·**POPC supported lipid bilayer interface.11*d* However, we have no concrete information about the position of the cavitand in the PC_m_ aggregate, so there is little hard evidence for this theory. The other possibility is that the lipids act as a “compression sleeve”, forcing the cavitand walls closer to the guest than normally observed in pure water. Cavitand **1** is highly flexible, and the exact position of the walls varies with guest size. A restricted “breathing” motion of the host walls would strengthen intermolecular host:guest interactions, as has been seen for numerous other encapsulation complexes, in water and in organic solvents.3a,b,^23^

**Fig. 4:**
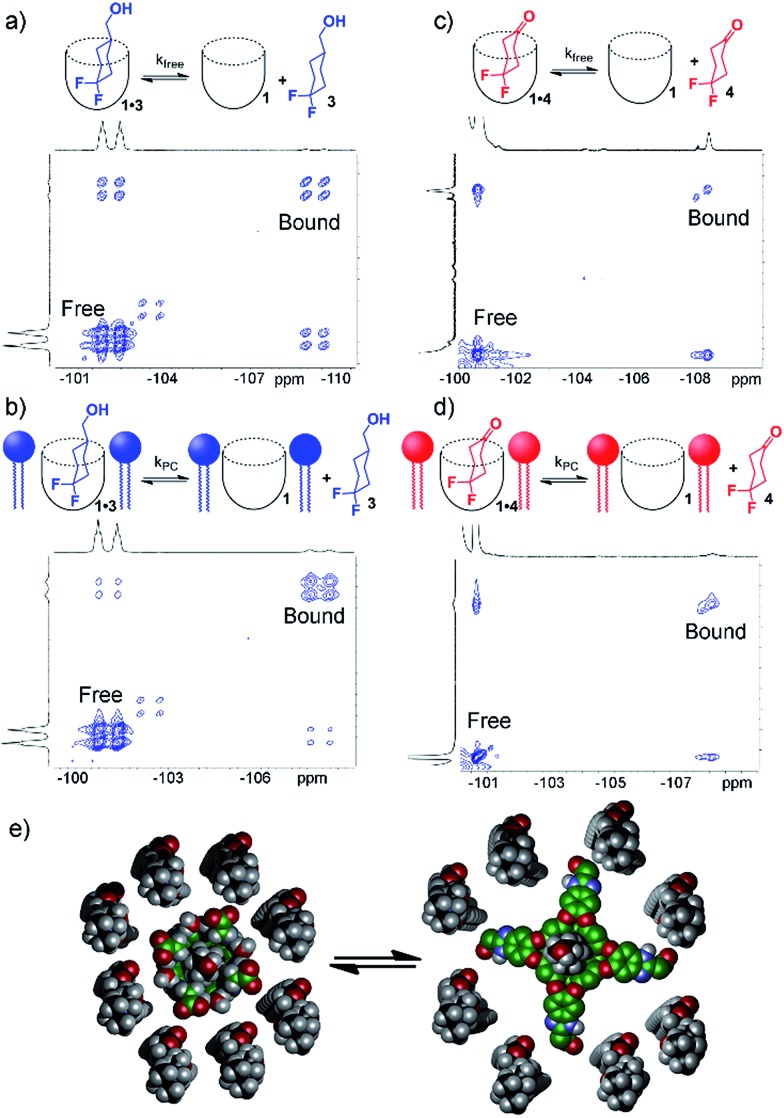
In/out exchange of guests **3** and **4** in **1** and **1·PC_m_**. ^19^F–^19^F EXSY NMR spectra at mixing time *τ* = 100 ms of (a) **1·3** in D_2_O solution; (b) **1·3·PC_m_**; (c) **1·4** in D_2_O solution; (d) **1·4·PC_m_** (376.50 MHz, 298 K, [**1**] = 5.8 mM, [**3**,**4**] = 39.5 mM, ratio DMPC/DHPC = 3.2 : 1, 60 mg mL^–1^ total lipid concentration); (e) representation of the exchange dynamics in cavitand 1 in a DMPC/DHPC lipid bilayer environment.

To shed light on this, as well as to gain valuable insight on the host:guest kinetics, we investigated the in/out exchange properties of **1** with guests **3** and **4** in solution and in the PC_m_ aggregate. NMR experiments that take advantage of magnetization transfer are ideally suited to kinetic analysis of host:guest systems.^24^ 2D ^1^H–^1^H EXSY experiments were previously used to show the exchange rates of small guests in and out of **1** in aqueous solution,2*a* but the presence of large interfering peaks from the lipids limits the effectiveness of these experiments. The presence of guest nuclei not present in the lipid aggregates allows exchange analysis, however, and 2D ^19^F–^19^F EXSY proved effective for kinetic analysis of guests **3** and **4**. Fig. 4 shows partial ^19^F–^19^F EXSY spectra for the **1·3** and **1·4** complexes, obtained under the same conditions as the spectra in Fig. 2 and 3. The major diagonal peak corresponds to the signals from the free and bound axial F in each molecule. At a mixing time of *τ* = 100 ms, exchange crosspeaks are easily observed, illustrating the in/out exchange process. No crosspeaks are seen at *τ* = 3 ms. The spectra in Fig. 4 clearly illustrate the qualitative differences in exchange behavior of **1·3** and **1·4** in D_2_O and in PC_m_. Whereas exchange crosspeaks are clearly visible at *τ* = 100 ms for **1·3** (D_2_O), the same exchange conditions show only minimal crosspeaks for **1·3·PC_m_** (Fig. 4a and b). Only at longer mixing times are crosspeaks observed, indicating a substantial slowing of the exchange rate of **3** when **1** is embedded in the PC_m_ aggregate. A similar, although less obvious effect is seen for guest **4**: exchange crosspeaks are smaller in the **1·4·PC_m_** system than in **1·4** at 100 ms (Fig. 4c and d).

By taking the exchange spectra at multiple different mixing times, quantitation of the exchange rates was possible. The EXSY spectra of **1·3** and **1·4** were surprisingly complex. Fig. 4 shows the relevant sections of the ^19^F–^19^F EXSY spectra (at mixing time *τ* = 100 ms) used to determine exchange rates; the full spectra are shown in the ESI.† Multiple peaks are observed in the full spectra: the axial and equatorial F atoms both show free and bound peaks, which show chemical exchange with each other. In addition, the geminal fluorines show NOE crosspeaks to each other, and small peaks are present from the other conformer with CH_2_OH axial. As such, the in/out exchange rates were determined by fitting the intensity of the four exchange peaks shown in Fig. 4 (obtained by extracting 1D slices from the 2D EXSY plots) against mixing time. At higher mixing times (*τ* ≥ 300 ms), the multiple methods of magnetization transfer in the system caused inaccuracies in the fitting, so the initial rate regions of the plot were used. For a detailed description of the fitting method, please see ESI.†

Table 1 shows the results of the exchange analysis. The fitting process gives the rate *k*_–1_ (or “*k*_off_”) for each guest, obtained at identical concentrations and temperatures for each guest in either aqueous solution (*k*_free_) or in the micelle environment (*k*_PC_). Eyring analysis^25^ of the rate constants gives the exchange barriers Δ*G*^‡^. As expected, the rate is dependent on the nature of the guest, but most interestingly, it is also dependent on the external environment. The larger guest **3** shows a *k*_free_ = 4.2 s^–1^, comparable to that previously obtained for cyclooctanol.2*a* In the presence of lipids, however, the exchange rate drops by over a factor of two, with *k*_PC_ = 1.8 s^–1^, corresponding to an additional 0.5 kcal mol^–1^ additional barrier conferred by the external environment surrounding the cavitand host. The same “compression sleeve” effect that enhanced the axial conformation of bound **3** slows the in/out exchange rate as well.

**Table 1:** Exchange rates and barriers for guest exchange in cavitand **1** in both free solution and DHPC/DMPC lipid aggregates^*a*^

Guest	*k*_free_, s^–1^	*k*_PC_, s^–1^	Δ*G*‡free, kcal mol^–1^	Δ*G*‡PC, kcal mol^–1^
**3**	4.2 ± 0.9	1.8 ± 0.8	16.6	17.1
**4**	8.7 ± 1.3	5.2 ± 1.6	16.2	16.5
**4_hyd_**	5.7 ± 0.8	N/A	16.4	N/A
**6**	N/A	3.0 ± 0.2	N/A	16.7
**7**	N/A	5.7 ± 0.5	N/A	16.4

^*a*^Exchange rates determined by fitting 2D EXSY crosspeaks (see ESI for fit plots and model). *k*_free_ = “off” exchange rate *k*_–1_ of guest from **1** in D_2_O. *k*_free_ = “off” exchange rate *k*_–1_ of guest from **1·PC_m_** in 1 mM HEPES/D_2_O, ratio DMPC/DHPC = 3.2 : 1, 60 mg mL^–1^ total lipid concentration. [**1**] = 5.8 mM, [**3**,**4**] = 39.5 mM, [**6**,**7**] = 16 mM. Exchange barriers determined *via* the Eyring equation.^25^

**Fig. 5:**
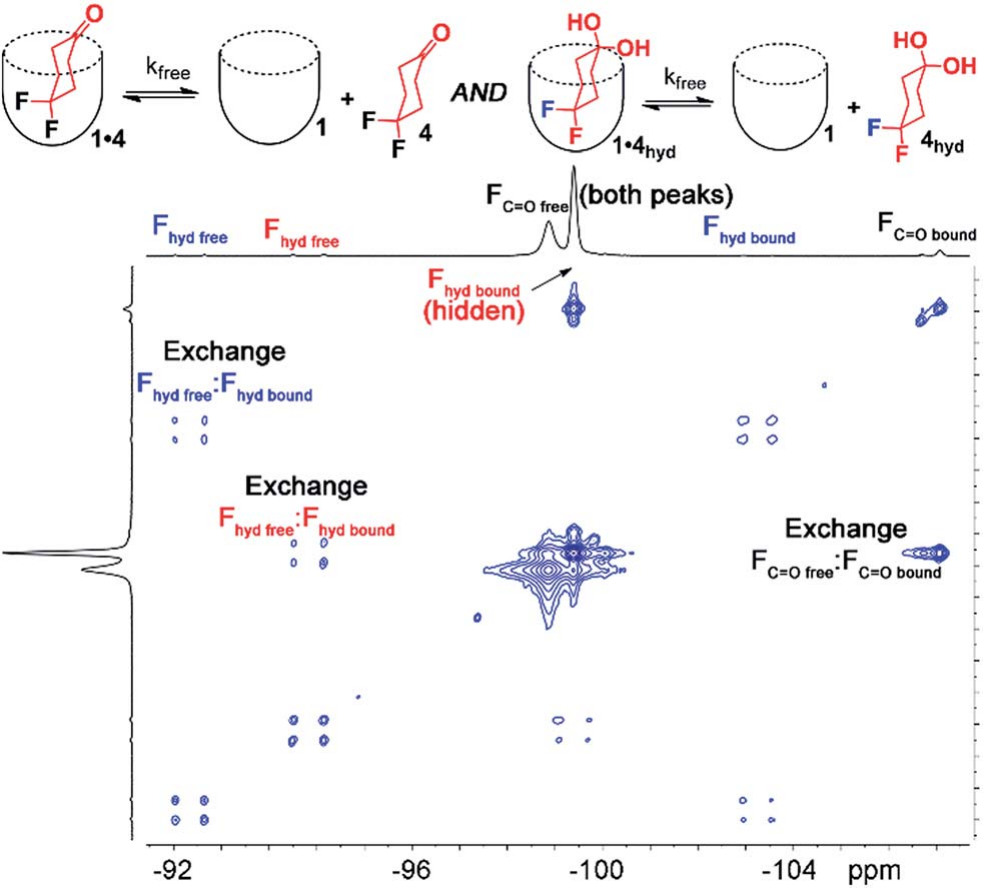
Full ^19^F EXSY spectrum of the cavitand **1·** guest **4** complex in pure D_2_O with peak assignments (D_2_O, 150.84 MHz, 298 K, mixing time = 150 ms, [**1**] = 5.8 mM, [**4**] = 39.5 mM).

EXSY analysis of guest **4** showed that *k*_free_ = 8.7 s^–1^, similar to that to that previously obtained for cyclohexanone, as expected.2*a* In a lipid environment, the exchange rate slowed again, with *k*_PC_ = 5.2 s^–1^. The retardation of exchange rate is slightly less in the case of the smaller guest **4**, with a 0.3 kcal mol^–1^ additional barrier. Surprisingly, the EXSY spectrum of **1·4** allowed analysis of the in/out exchange of the hydrated *gem*-diol form of **4_hyd_**, as the crosspeaks were large enough to observe (Fig. 5). The additional hydrogen bonding present in **4_hyd_** slows the exchange rate when compared to the ketone form, and *k*_free_ (**4_hyd_**) = 5.7 s^–1^. Unfortunately, the equivalent crosspeaks in the PC_m_ system were too small to accurately fit, so determination of *k*_PC_ was unsuccessful in that case.

The use of guest nuclei other than ^1^H to analyze the in/out exchange allows analysis of other, more biorelevant guests such as choline. While association constants for R-NMe_3_^+^ guests are easily determinable by ITC10*b* or indicator displacement assays,^13^ NMR analysis of the exchange kinetics are complicated by self-aggregation. Cavitand **1** is susceptible to aggregation in free solution in the presence of lipophilic salts such as choline. Stable 1 : 1 complexes can be observed by NMR when substoichiometric amounts of guest are used,10*b* but addition of excess RNMe_3_^+^ salt causes aggregation and peak broadening, limiting analysis of the in/out rate by exchange NMR.^13^ This does not occur in lipid environments, rendering this system ideal for analyzing exchange of hydrophilic, yet strongly binding salts **5–8**. ^13^C-enriched R-NMe_3_^+^ guests were easily accessed *via* reaction of the corresponding dimethylamino precursor with enriched ^13^CH_3_I. As cavitand **1** is capable of binding numerous R-NMe_3_^+^ salts in lipid environment, we investigated a series of guests **5–8** with variable upper rim functionality. The bola-type bis-NMe_3_^+^ guests **5** and **8** were initially targeted to investigate the possibility of slowed tumbling in the cavity of **1**, as opposed to in/out guest exchange. Encapsulation in **1** slows the up/down interconversion rate of hydrocarbons such as *trans*-decalin, but quantitation is challenging due to peak broadening. As **5** and **8** are symmetrical, it was envisaged that exchange would be observed between the two conformers in the **1·5·PC_m_** system *via*^13^C–^13^C EXSY NMR analysis. Unfortunately, neither guest **5** nor **8** are bound by **1** in either free aqueous solution or in the PC_m_ environment. Evidently, the nature of the upper rim has a large effect on guest recognition, so we turned to guests that can display favorable H-bonds with the carboxylate groups, choline **6** and the dimethylamino-variant **7**.

**Fig. 6:**
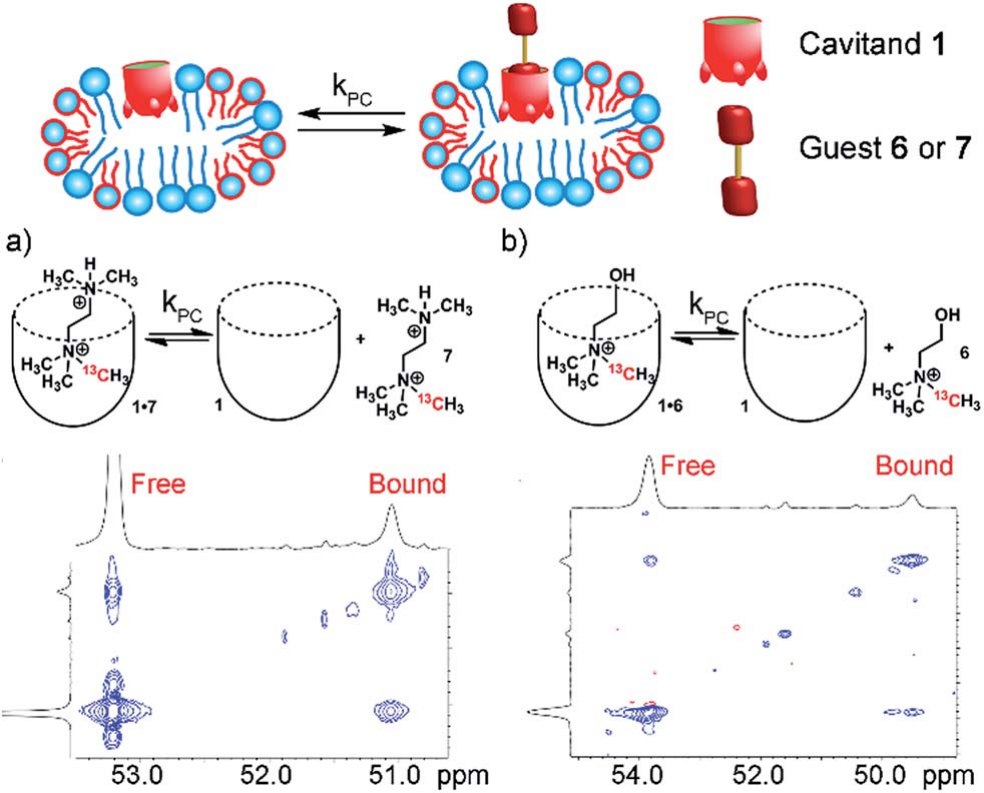
In/out exchange of guests **6** and **7** in **1** and **1·PC_m_**. ^13^C–^13^C EXSY NMR spectra at mixing time *τ* = 100 ms of (a) **1·7·PC_m_**; (b) **1·6·PC_m_** (2.5 mM HEPES/D_2_O, 150.84 MHz, 298 K, [**1**] = 5.8 mM, [**6**,**7**] = 16 mM, ratio DMPC/DHPC = 3.2 : 1, 60 mg mL^–1^ total lipid concentration).

Analysis of the unsymmetrical guests **6** and **7** was far more successful, and representative examples of the ^13^C–^13^C EXSY spectra are shown in Fig. 6. The peak for free R-NMe_3_^+^ guest (**6** or **7**) overlaps with peaks from the R-NMe_3_^+^ group in the phosphocholine lipids, as would be expected. Even though **6**/**7** are ^13^C-enriched, a significant proportion of ^13^C-PC is present due to the excess of lipids in the sample. Despite the interfering peaks for free guest, ^13^C peaks for both bound **1·6** and **1·7** are easily observable, with the characteristic upfield shift observed. The relative change in ^13^C *δ* upon binding is proportionally smaller than that for ^1^H, with Δ*δ* ∼ –2 ppm, but this is easily enough to allow exchange analysis *via*^13^C–^13^C EXSY.

^13^C–^13^C EXSY spectra with varying mixing times were obtained for samples of **1·6·PC_m_** and **1·7·PC_m_** at 298 K with 5.8 mM **1**, 16 mM guest and 60 mg mL^–1^ lipid, as usual. The exchange rates were acquired *via* fitting the crosspeak intensities extracted as slices from the 2D spectra. In this case, the diagonal peak corresponding to free guest overlapped with peaks from the NMe_3_^+^ groups in the lipids. As the concentration of the lipids was constant, the signal from the lipids remained constant in the low mixing time experiments, and the accuracy of the fit for the off-rate was not compromised. The rates are shown in Table 1. Interestingly, the rates are broadly similar to those observed with the difluorocyclohexanyl guests, with *k*_PC_ (**6**) = 3.0 s^–1^ and *k*_PC_ (**7**) = 5.7 s^–1^. The bulkier **7** exchanges more rapidly than choline **6**, in contrast to the results for hydrophobic guests, where larger guests showed slowed exchange. It is likely that positioning the extra steric bulk at the upper rim lowers the affinity of **7** for the cavitand, as has been observed for other R-NMe_3_^+^ species, and a more rapid exchange rate is seen. The results from Table 1 also allow an estimate of the *k*_free_ for **6** and **7**: if the “compression sleeve” effect of the micelle environment is assumed to be constant, then *k*_free_ for **6** and **7** would be on the order of ∼6 s^–1^ and ∼10 s^–1^, respectively.

**Fig. 7:**
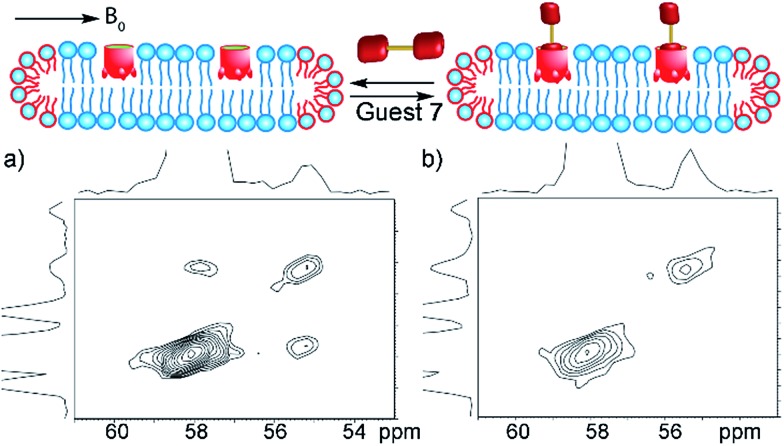
In/out exchange of guest **7** in the magnetically ordered bicelle system PC_b_. ^13^C–^13^C EXSY NMR spectra at mixing time (a) *τ* = 20 ms; (b) *τ* = 0 ms of **1·7·PC_b_**; (1 mM HEPES/D_2_O, 100.69 MHz, 298 K, [**1**] = 20 mM, [**7**] = 36 mM, ratio DMPC/DHPC = 3.2 : 1, 150 mg mL^–1^ total lipid concentration).

As the ^13^C-labeled guests **6** and **7** were amenable to EXSY analysis in isotropically tumbling micelles, we next employed these guests towards detection of in/out exchange of host **1** in the more challenging, yet more relevant magnetically ordered bicelles. The bicelles were formed as described above (also see Experimental), loaded into a 4 mm Bruker solid state rotor, and solutions of cavitand **1** and guests **6** and **7** were added. As might be expected, the larger concentration of lipids (and their overlapping phosphocholine groups) made analysis *via* 1D NMR challenging, even with ^13^C-enriched guests. Fortunately, the magnetic alignment at 308 K was good, and the presence of exchanging guest **7** was observable by ^13^C–^13^C EXSY analysis (see Fig. 7a). The signal : noise ratio was poor for ^13^C choline **6**, so we focused on guest **7** for bicellar analysis. At mixing time *τ* = 20 ms, exchanging crosspeaks corresponding to the ^13^CH_3_ signal from bound and free guest **7** can be seen. At the elevated temperatures required for magnetically ordered bicelle formation, the in/out rate occurs more rapidly, and a shorter mixing time was needed to see exchange. The nature of the crosspeaks was corroborated by the EXSY spectrum taken with mixing time *τ* = 0 ms (Fig. 7b), where no crosspeaks could be seen. Unfortunately, accurate quantitation of the exchange rate in the bicellar system proved challenging. The smaller sample volume necessitated a greater amount of signal averaging to obtain good signal, and required long acquisition times (∼48 h per spectrum). In addition, the bicelles decomposed after ∼1 week at 308 K. The experiments were performed on the same sample to avoid differences in peak intensity due to any slight differences in concentration between samples, and to obtain spectra in manageable timeframes, the resolution in the F2 dimension was reduced. As a result, the spectra were suitable for only qualitative analysis rather than quantitation of the exchange rate. However, the exchange could be clearly seen for guest **7**, and illustrates the power of the system: 2D NMR analysis of the host:guest properties of cavitand **1** is possible in different types of complex lipid aggregates, and the guest kinetics can be analyzed for guests containing suitable nuclei for detection.

## Conclusions

By employing guests with detectable nuclei, NMR analysis of how the external environment affects the recognition properties of a water-soluble deep cavitand is possible. Nuclei such as ^13^C or ^19^F are usually not used to analyze conformation and motion of small molecules in confined environments, as the chemical shift changes are small relative to ^1^H and lone-pair containing groups can lower affinity, especially for aromatic hosts. The systems studied here illustrate a wide variety of effects that can be conferred on a small molecule guest from molecules outside the host. Embedding the deep cavitand in lipids compresses the flexible walls of the host, enhancing its recognition properties and providing an additional barrier to wall-opening. By forcing the walls of the closer to the guest, unfavorable conformational or reaction equilibria can be enhanced: favorable H-bonding with the upper rim carboxylates enhances the population of an axial cyclohexyl conformer, as well as favoring ketone hydration. These unusual conformations are present for the host:guest complexes in water, but are enhanced in the lipid environment. In addition, 2D EXSY NMR spectroscopy using either ^13^C or ^19^F as detectable nucleus allows analysis of the in/out exchange properties of bound guests in phosphocholine lipid micelles and magnetically ordered bicelles. Embedding the host in a lipid aggregate slows the exchange rate of small molecule guests by over a factor of two, due to the energetic penalty conferred on the opening of the host walls by the external lipid aggregate. Both solution- and solid-state NMR techniques were employed to show this exchange process, providing the first detailed view of the exchange process of flexible supramolecular host molecules in biomimetic lipid membranes. Further studies of molecular recognition in complex systems is underway in our laboratory.

## Experimental

### General information

1D NMR experiments (^1^H, ^13^C, ^19^F) were performed on a Bruker Avance NEO 400 9.4 T spectrometer with a 5 mm Prodigy CPP BBO BB-H&F z-gradient cryo-probe or a Bruker 14.1 T (600.01 MHz ^1^H) Avance I spectrometer equipped with a 5 mm BBO Z-grad probe. Micelle (PC_m_) experiments (^1^H–^1^H COSY, ^2^H–^2^H EXSY, ^13^C–^13^C EXSY, ^19^F–^19^F EXSY) were performed on a Bruker Avance NEO 400 9.4 T spectrometer with a 5 mm Prodigy CPP BBO BB-H&F z-gradient cryo-probe, a Bruker 14.1 T (600.01 MHz ^1^H) Avance I spectrometer equipped with a 5 mm BBO Z-grad probe or a Bruker Avance III 700 16.44 T spectrometer with a 5 mm CP TCI H–C/N-D z-gradient cryo-probe. Magnetically ordered bicelle (PC_b_) experiments (^13^C, ^13^C–^13^C EXSY) were performed at 9.4 T (400.37 MHz ^1^H, 100.69 MHz ^13^C, 162.07 MHz ^31^P) on a Bruker AVIII spectrometer equipped with a double resonance, 4 mm MAS probe. Proton (^1^H) and carbon (^13^C) chemical shifts are reported in parts per million (*δ*) with respect to tetramethylsilane (TMS, *δ* = 0). Phosphorus (^31^P) chemical shifts are reported in parts per million (*δ*), and referenced internally with respect to 85% H_3_PO_4_. Fluorine (^19^F) chemical shifts are reported in parts per million (*δ*), and referenced internally with respect to CF_3_COOH. Deuterated NMR solvents were obtained from Cambridge Isotope Laboratories, Inc., Andover, MA, and used without further purification. Mass spectra were recorded on an Agilent 6210 LC TOF mass spectrometer using electrospray ionization with fragmentation voltage set at 115 V and processed with an Agilent MassHunter Operating System. All other materials (including guests **2–4**, synthetic precursors for guests **5–8**, dimyristoylphosphocholine (DMPC), and diheptylphosphocholine (DHPC)) were obtained from Aldrich Chemical Company, St. Louis, MO, or TCI, Tokyo, Japan and were used as received. Solvents were dried through a commercial solvent purification system (Pure Process Technologies, Inc.). Molecular modeling (molecular mechanics calculations) was carried out using the AMBER force field^26^ with the solvation (dielectric) setting for water as implemented by SPARTAN. Cavitand **1** was synthesized according to published procedures:10*b* also see this paper for the NMR spectra of the **1·** choline complex.

### Experimental procedures

#### Micelle (PC_m_) preparation

Mixed lipid micelles with *q* = 3.2 (*q* = long chain lipid/short chain lipid) were formed by mixing together DHPC and DMPC dissolved in chloroform. The chloroform was evaporated off under a stream of nitrogen and then the lipids were lyophilized for 4 h before the micelles were prepared to remove any residual TFA and chloroform. The solid lipids were dissolved in HEPES buffer, pH = 6.5 for a combined lipid concentration of 290 mg mL^–1^. NMR samples were prepared by dissolving 100 μL of the lipid mixture in 400 μL D_2_O, to a final combined lipid concentration of 60 mg mL^–1^, 1 mM HEPES. Cavitand **1** and guests were added to the mixture and equilibrated for 1 h before NMR analysis.

#### Ordered bicelle (PC_b_) preparation

Magnetically ordered bicelles were prepared according to literature procedures.^27^,^28^ The DMPC/DHPC lipid mixture was made with *q* = 3.2 (*q* = long chain lipid/short chain lipid) by mixing together DHPC and DMPC dissolved in chloroform. The chloroform was evaporated off under a stream of nitrogen, the lipids were lyophilized for 4 hours before the bicelles were prepared to remove any residual TFA and chloroform, and the solid lipids were dissolved in HEPES buffer, pH = 6.5 for a combined lipid concentration of 290 mg mL^–1^. This solution was subjected to 5 minutes of vortexing, followed by 30 minutes in a water bath at 45 °C, followed by 15 minutes in an ice bath. This process was repeated five times until the lipids were clear and non-viscous at 4 °C and milky-white and solid at room temperature, indicative of bicelle formation. NMR samples were prepared by dissolving 250 μL of the bicelles in 200 μL HEPES buffer, pH = 6.5, and 50 μL D_2_O for a lock, to a final combined lipid concentration of 150 mg mL^–1^, 2.5 mM HEPES. This solution was added to a 4 mm Bruker solid-state rotor. Cavitand **1** and guests were added to the mixture and equilibrated for 24 h before NMR analysis.

#### Procedure for 2D solution-phase EXSY experiments

The 2D NOESY spectra of the cavitand:guest exchange processes were recorded at 298 K at either 400 MHz or 600 MHz with the phase sensitive ^19^F–^19^F or ^13^C–^13^C NOESY pulse sequence supplied with the Bruker software. Each of the 512 F1 increments was the accumulation of 6 scans (^19^F) or 32 scans (^13^C). Before Fourier transformation, the FIDs were multiplied by a 90° phase-shifted sine square function in both the F2 and the F1 domain. 1 K _ 1 K real data points were used, with a resolution of 1 Hz per point.

#### Procedure for 2D bicelle EXSY experiments

Bicelle samples containing guest 7 and cavitand were loaded in a 4 mm Bruker solid-state rotor, and experiments were performed on a Bruker AVIII spectrometer equipped with a ^1^H-X double resonance 4 mm MAS probe with no spinning. The 2D EXSY spectra of the cavitand:guest 7 exchange process in bicelles were recorded at 316 K at 9.4 T with a NOESY pulse sequence. Each of the 128 F1 increments was the accumulation of 128 scans with a relaxation delay of 3 s. Before Fourier transformation the FID was multiplied by a 90° sine square function in the F1 domain and an EM function in the F2 domain. 2 K (F2) _ 256 (F1) real data points were used with a resolution of 1 Hz per point.

### Synthesis of new molecules

#### *N*,*N*,*N*,*N*′,*N*′,*N*′-Hexamethyl-1,3-propyldiaminium diiodide-^13^C **5**

Tetramethyl-1,3-diaminopropane (0.1 mL, 0.6 mmol) was dissolved in 2 mL of acetonitrile at 0 °C followed by addition of iodomethane-^13^C (0.09 mL, 1.32 mmol) dropwise. The reaction was stirred at ambient temperature for 16 hours, and the resulting precipitate was collected by vacuum filtration to obtain 220 mg of guest **5** as a white solid (88% yield). ^1^H NMR (400 MHz, D_2_O) *δ* 3.47 (m, 4H), 3.23 (t, *J* = 145 Hz, 18H, ^13^C–^1^H coupling) 2.41 (m, 2H), ^13^C NMR (100 MHz, D_2_O) *δ* 62.3, 53.3, 17.4. ESI-MS *m*/*z* expected: 190.20, found [MH^+^] = 190.14.

#### Choline-^13^C iodide **6**

Dimethylethanolamine (107 mg, 1.2 mmol) dissolved in 1 mL of acetonitrile at 0 °C followed by addition of iodomethane-^13^C (0.1 mL, 1.2 mmol) dropwise. The reaction was stirred at ambient temperature for 16 hours, and the resulting precipitate was collected by vacuum filtration to obtain 228 mg of a white solid (82% yield). ^1^H NMR (400 MHz, D_2_O) *δ* 3.92 (t, 2H), 3.39 (t, *J* = 145 Hz, 2H), 3.07 (s, 9H). ^13^C NMR (125 MHz, D_2_O) *δ* 67.4, 55.6, 53.9. ESI-MS *m*/*z* expected: 105.16, found [M^+^] = 105.11.

#### *N*,*N*,*N*,*N*′,*N*′-Pentamethyl-1,2-ethanediaminium iodide-^13^C **7**

Tetramethylethylenediamine (0.5 mL, 3.3 mmol) was dissolved in 1 mL of acetonitrile at 0 °C followed by addition of iodomethane-^13^C (0.2 mL, 3.3 mmol) dropwise. The reaction was stirred at ambient temperature for 16 hours and the resulting precipitate was collected by vacuum filtration to obtain 650 mg of white solid (76% yield). ^1^H NMR (400 MHz, D_2_O) *δ* 3.35 (t, 2H), 3.02 (t, *J* = 145 Hz, 9H, ^13^C–^1^H coupling), 2.73 (m, 2H), 2.16 (s, 6H), ^13^C NMR (125 MHz, D_2_O) *δ* 53.8, 53.2, 44.1. ESI-MS *m*/*z* expected: 132.23, found [MH^+^] = 132.16.

#### *N*,*N*,*N*,*N*′,*N*′,*N*′-Hexamethyl-1,2-ethanediaminium diiodide-^13^C **8**

*N*,*N*,*N*,*N*′,*N*′-Pentamethyl-1,2-ethanediaminium iodide-^13^C **7** (300 mg, 1.15 mmol) was dissolved in 1 mL of acetonitrile at 0 °C followed by addition of iodomethane-^13^C (0.1 mL, 1.2 mmol) dropwise. The reaction was stirred at ambient temperature for 16 hours, and the resulting precipitate was collected by vacuum filtration to obtain 256 mg of a white solid (56% yield). ^1^H NMR (400 MHz, D_2_O) *δ* 4.09 (t, 4H), 3.35 (t, *J* = 145 Hz, 18H, ^13^C–^1^H coupling), ^13^C NMR (125 MHz, D_2_O) *δ* 57.3, 53.7. ESI-MS *m*/*z* expected: 148.26, found [MH^+^] = 148.19.

## Conflicts of interest

There are no conflicts to declare.

## Supplementary Material

Supplementary information
