# Supplementary material for: Lipid bilayer environments control exchange kinetics of deep cavitand hosts and enhance disfavored guest conformations
Source: Chem Sci. 2018 Jan 11;9(7):1836–45. doi: 10.1039/c7sc05155g (PMC5890788; doi:10.1039/c7sc05155g)
Supplement: Supplementary file 1 [file SC-009-C7SC05155G-s001.pdf]

Electronic Supplementary Material (ESI) for Chemical Science.
This journal is © The Royal Society of Chemistry 2018

Lipid Bilayer Environments Control Exchange Kinetics of Deep Cavitand Hosts and Enhance Disfavored Guest Conformations

Lizeth Perez, Bethany G. Caulkins, Magi Mettry, Leonard J. Mueller and Richard J. Hooley*

Department of Chemistry, University of California-Riverside, Riverside, CA 92521, United States

richard.hooley@ucr.edu

Electronic Supplementary Information

Table of Contents

1. NMR Spectra of New Compounds S-2
2. NMR Spectra of Host:Guest Complexes in Free Aqueous Solution S-6
3. NMR Spectra of Host:Guest Complexes in DMPC/DHPC Lipid Micelles....................S-9
4. Fitting model for EXSY Experiments....................S-15
5. 2D Exchange NMR Spectra in Water and in DMPC/DHPC Lipid Micelles....................S-16
6. 2D Exchange NMR Spectra in Magnetically Ordered DMPC/DHPC Bicelles....................S-25

1. NMR Spectra of New Compounds

Guest 3

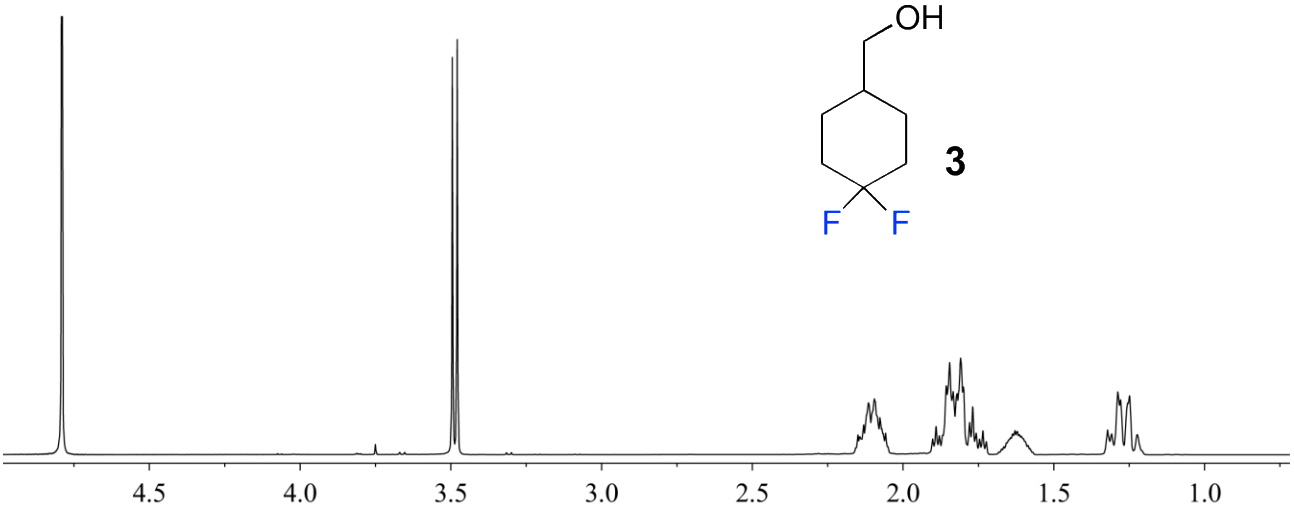

Figure S-1. ^{1}H NMR spectrum of 4,4-difluorocyclohexanemethanol guest **3** (400.13 MHz, D_2O, 298 K).

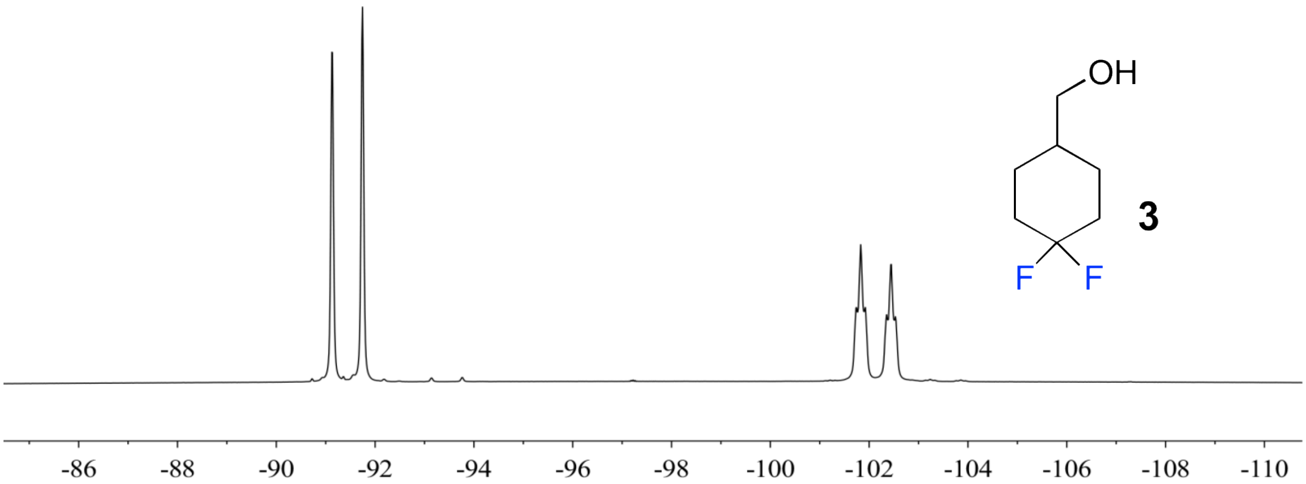

Figure S-2. ^{19}F NMR spectrum of 4,4-difluorocyclohexanemethanol guest **3** (376.50 MHz, D_2O, 298 K).

Guest 4

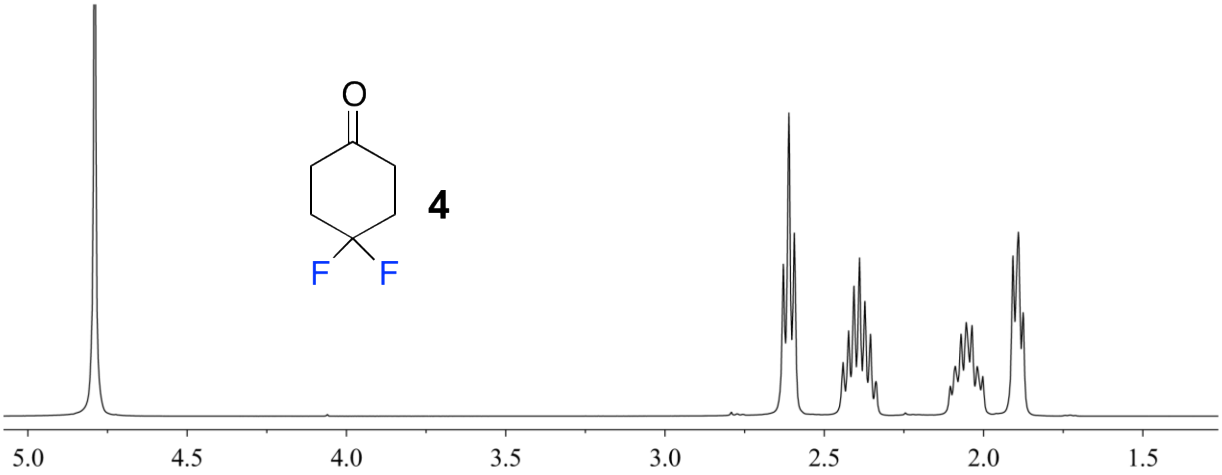

Figure S-3. ^{1}H NMR spectrum of 4,4-difluorocyclohexanone guest **4** (400.13 MHz, D_2O, 298 K).

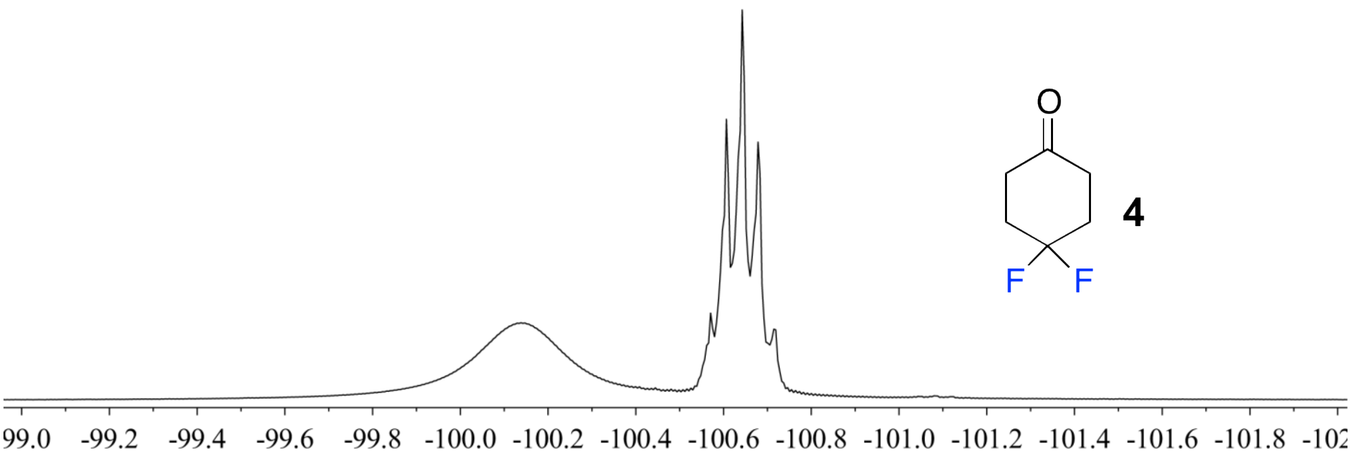

Figure S-4. ^{19}F NMR spectrum of 4,4-difluorocyclohexanone guest **4** (376.50 MHz, D_2O, 298 K).

Guest 5

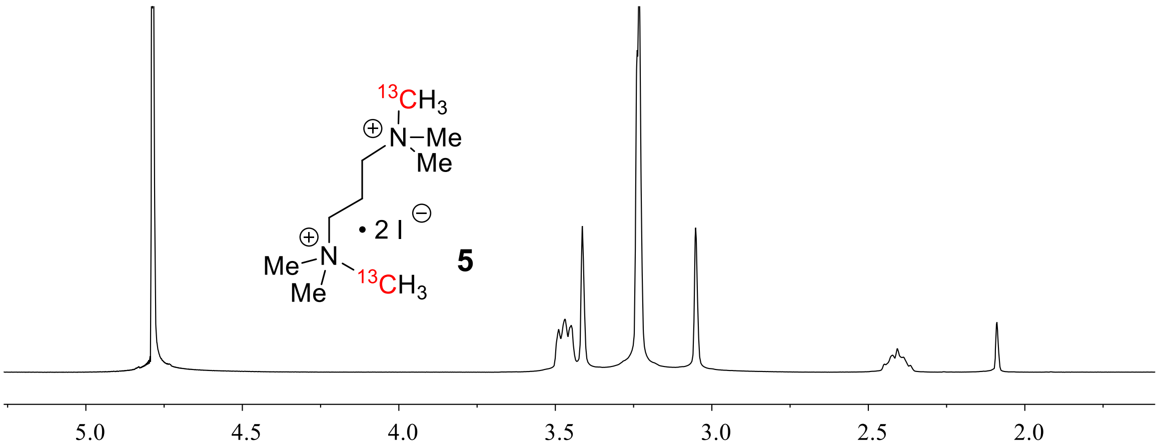

Figure S-5. ^{1}H NMR spectrum of N,N,N,N',N',N'-hexamethyl-1,3-propanediaminium diiodide-^{13}C guest **5** (400.13 MHz, D_2O, 298 K).

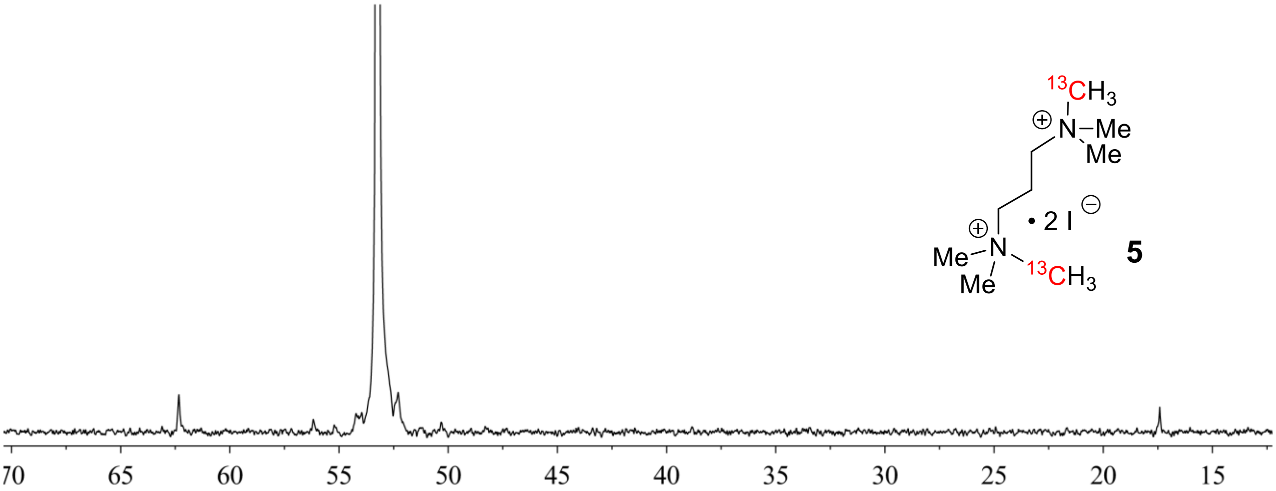

Figure S-6. ^{13}C NMR spectrum of N,N,N,N',N',N'-hexamethyl-1,3-propanediaminium diiodide-^{13}C guest **9** (100.61 MHz, D_2O, 298 K).

Guest 6

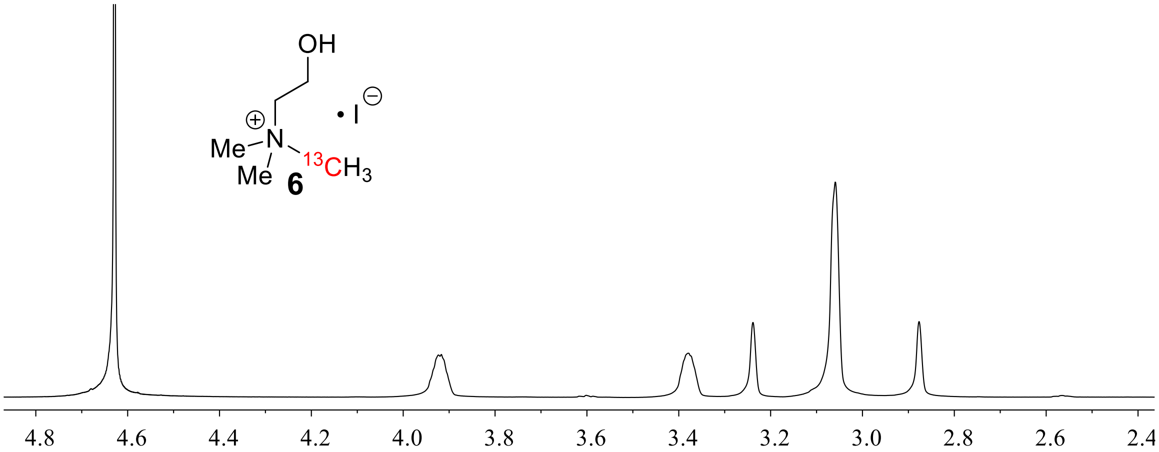

Figure S-7. ^{1}H NMR spectrum of choline iodide-^{13}C guest **6** (400.13 MHz, D_2O, 298 K).

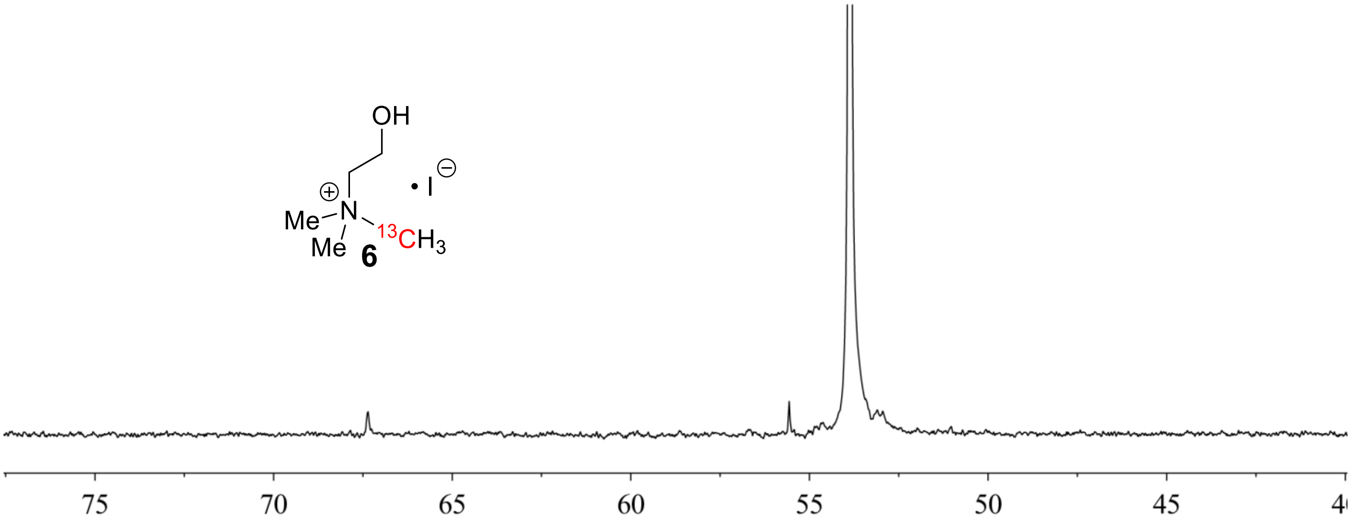

Figure S-8. ^{13}C NMR spectrum of choline iodide-^{13}C guest **6** (100.61 MHz, D_2O, 298 K).

Guest 7

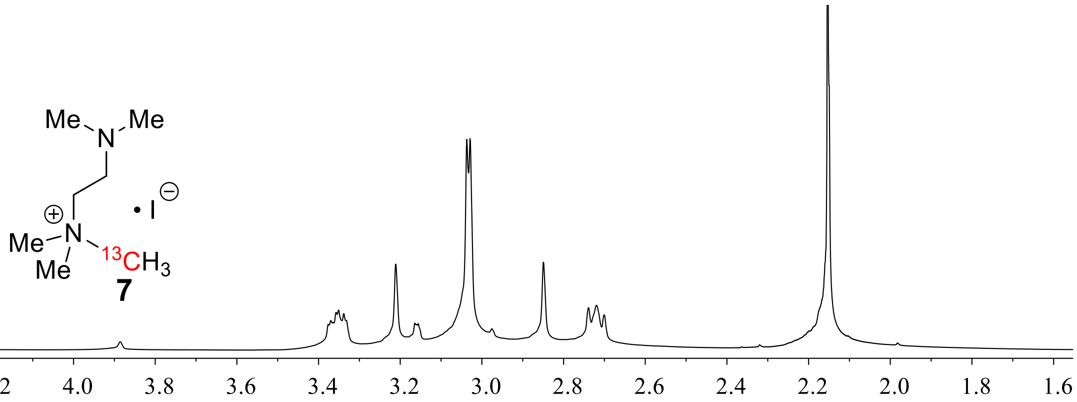

Figure S-9. ^{1}H NMR spectrum of N,N,N,N',N'-pentamethyl-1,2-ethanediaminium iodide-^{13}C guest **7** (400.13 MHz, D_2O, 298 K).

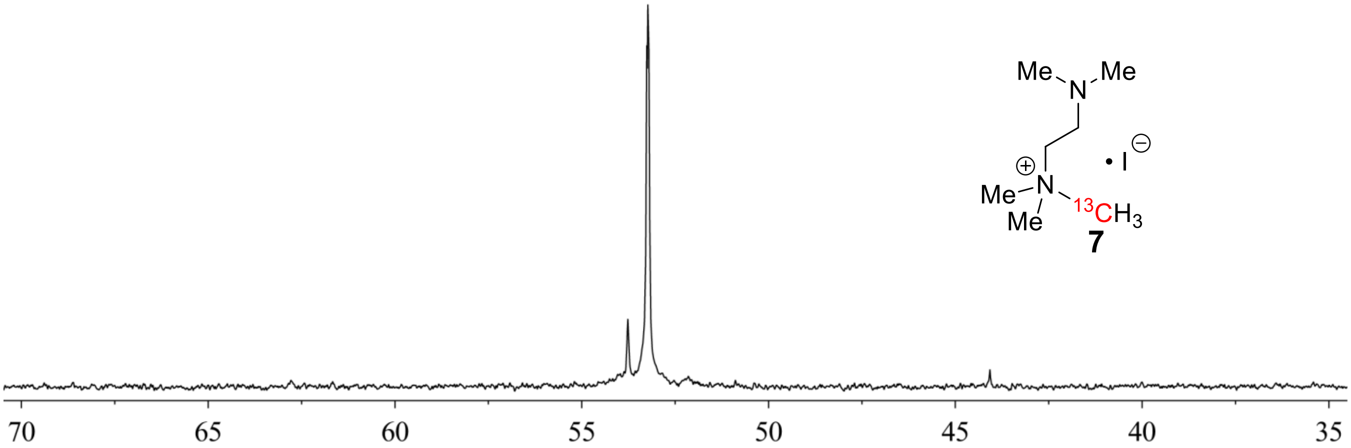

Figure S-10. ^{13}C NMR spectrum of N, N, N, N’, N’-pentamethyl-1,2-ethanediaminium iodide-^{13}C guest **7** (100.61 MHz, D_2O, 298 K).

Guest 8

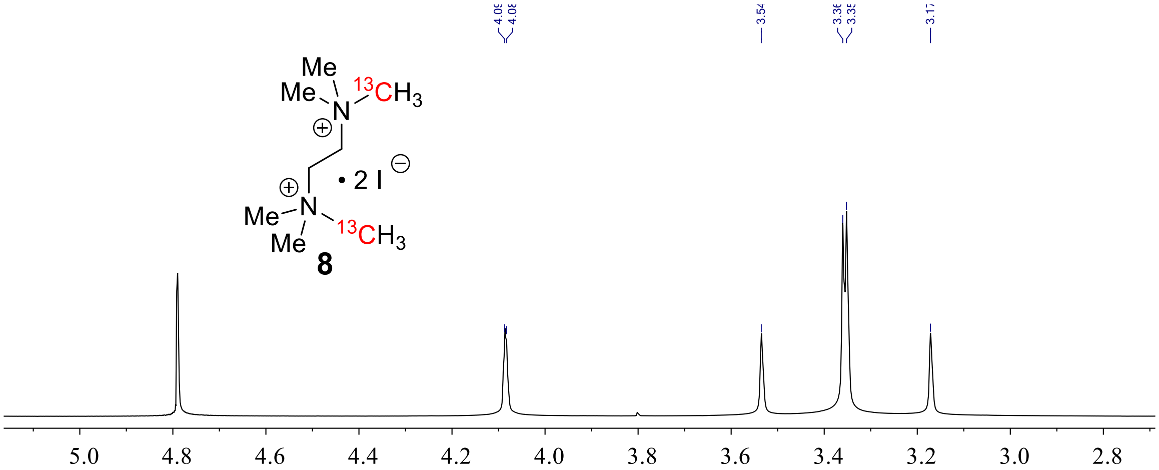

Figure S-11. ^{1}H NMR spectrum of N,N,N,N’,N’,N’-hexamethyl-1,2-ethanediaminium diiodide-^{13}C guest **8** (400.13 MHz, D_2O, 298 K).

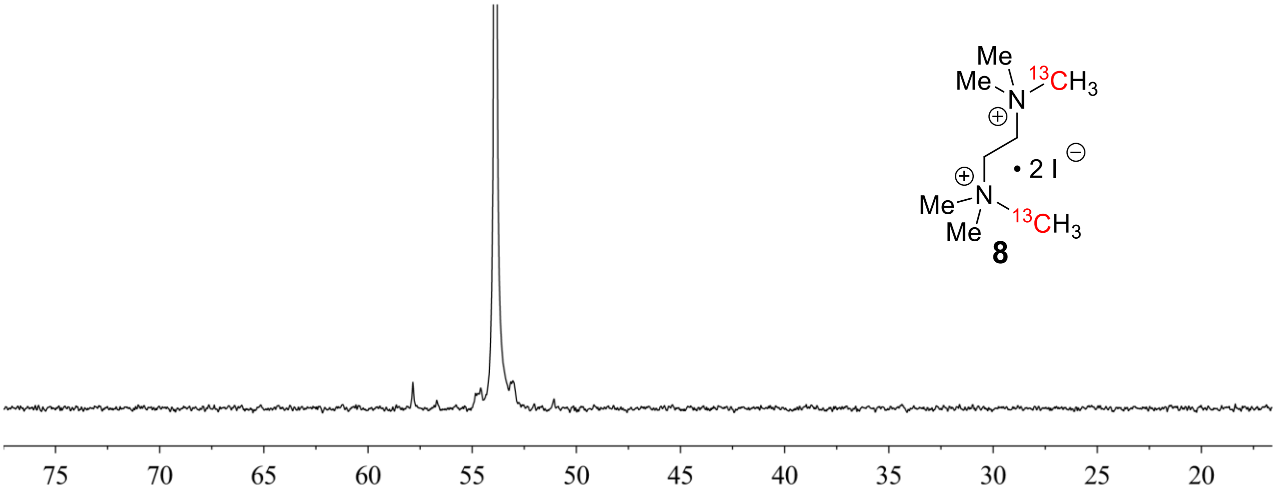

Figure S-12. ^{13}C NMR spectrum of N,N,N,N’,N’,N’-hexamethyl-1,2-ethanediaminium diiodide-^{13}C guest **8** (100.61 MHz, D_2O, 298 K).

2. NMR Spectra of Host:Guest Complexes in Free Aqueous Solution

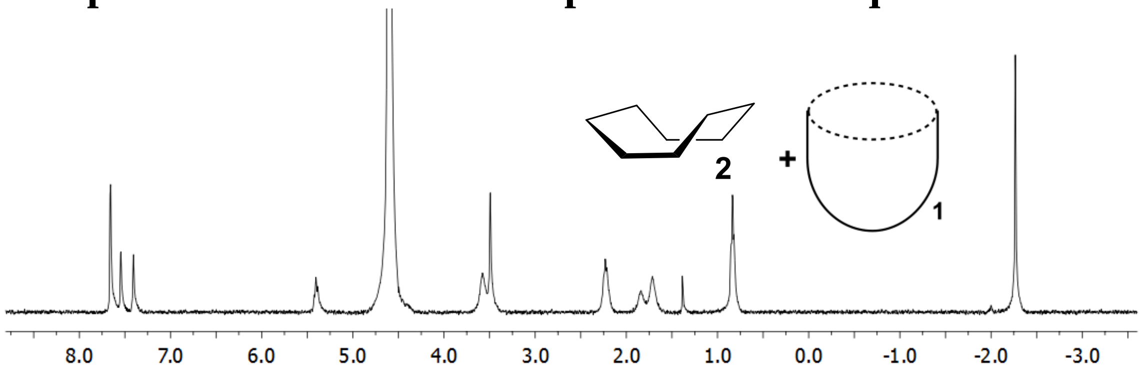

Figure S-13. ^{1}H NMR spectrum of the **1•2** cavitand-cyclooctane complex (400.13 MHz, D_2O, 298 K, [**1**] = [**2**] = 1.8 mM).

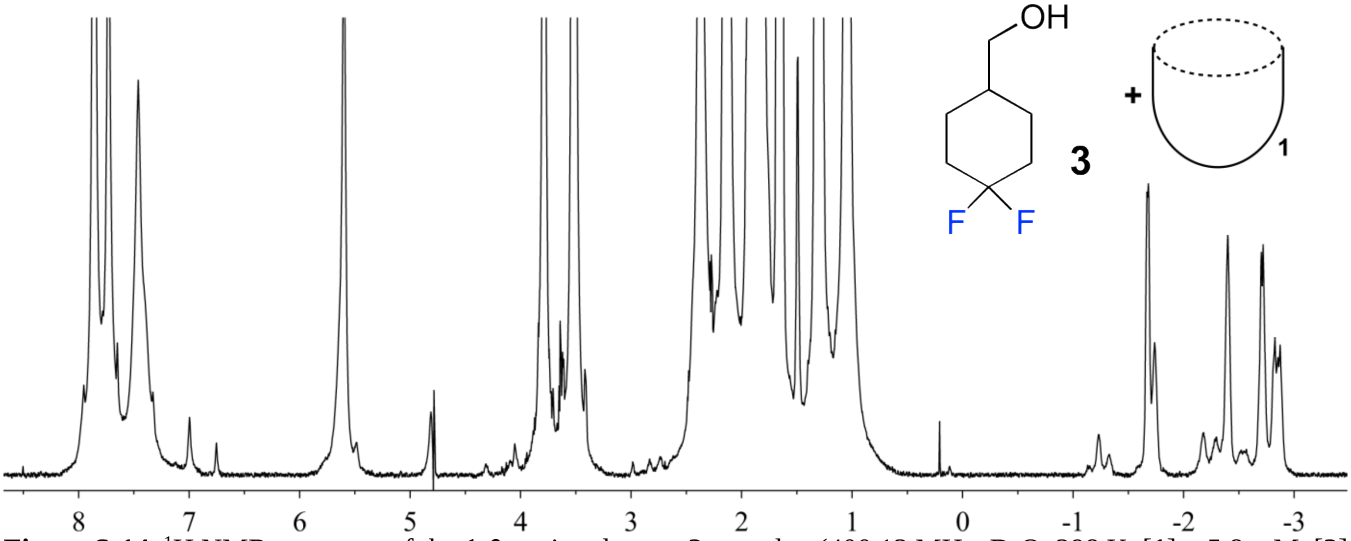

Figure S-14. ^{1}H NMR spectrum of the **1•3** cavitand-guest **3** complex (400.13 MHz, D_2O, 298 K, [**1**] = 5.8 mM, [**3**] = 39.5 mM).

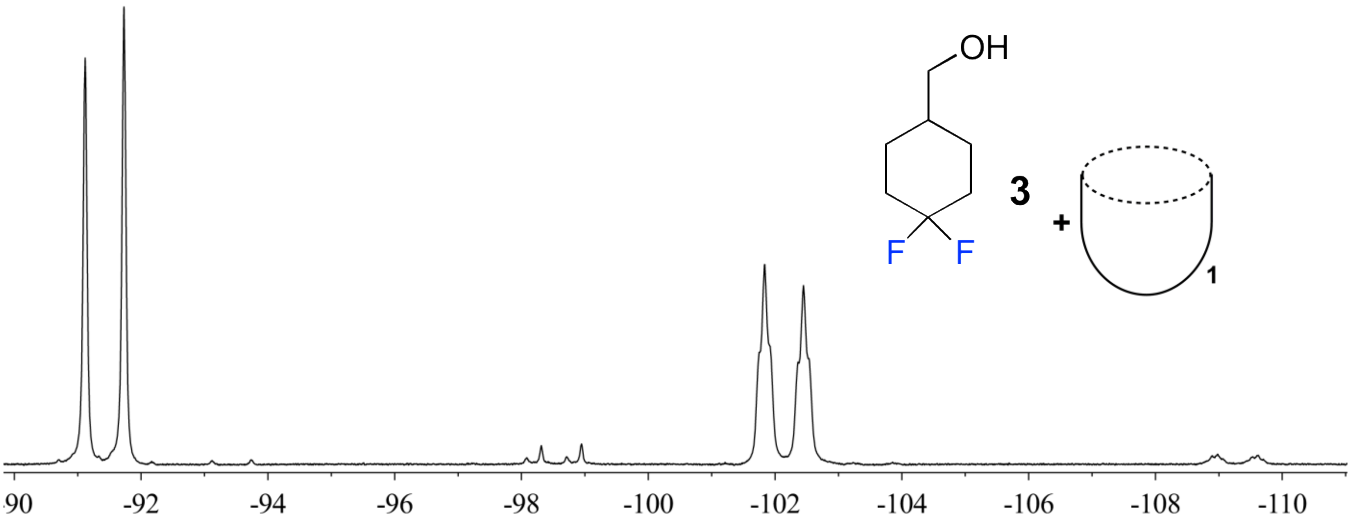

Figure S-15. ^{19}F NMR spectrum of the **1•3** cavitand-guest **3** complex (376.50 MHz, D_2O, 298 K, [**1**] = 5.8 mM, [**3**] = 39.5 mM).

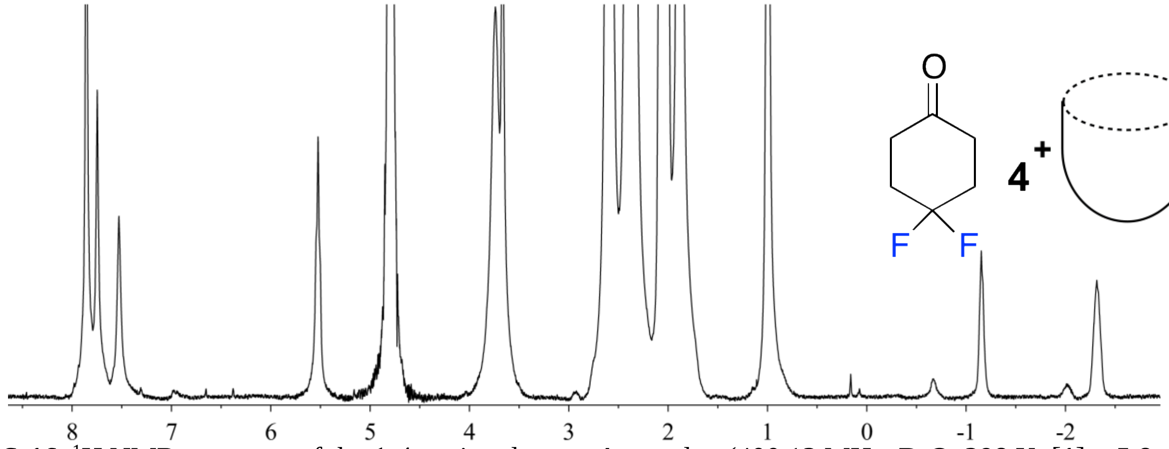

Figure S-16. ^{1}H NMR spectrum of the **1•4** cavitand-guest **4** complex (400.13 MHz, D_2O, 298 K, [**1**] = 5.8 mM, [**4**] = 39.5 mM).

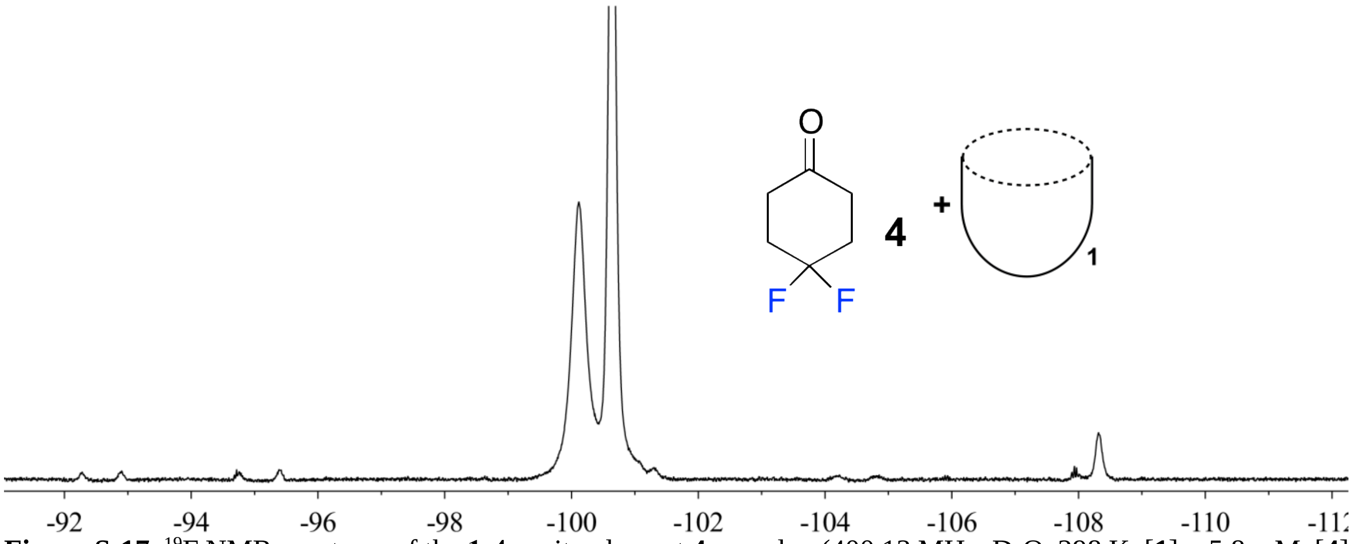

Figure S-17. ^{19}F NMR spectrum of the **1•4** cavitand-guest **4** complex (400.13 MHz, D_2O, 298 K, [**1**] = 5.8 mM, [**4**] = 39.5 mM).

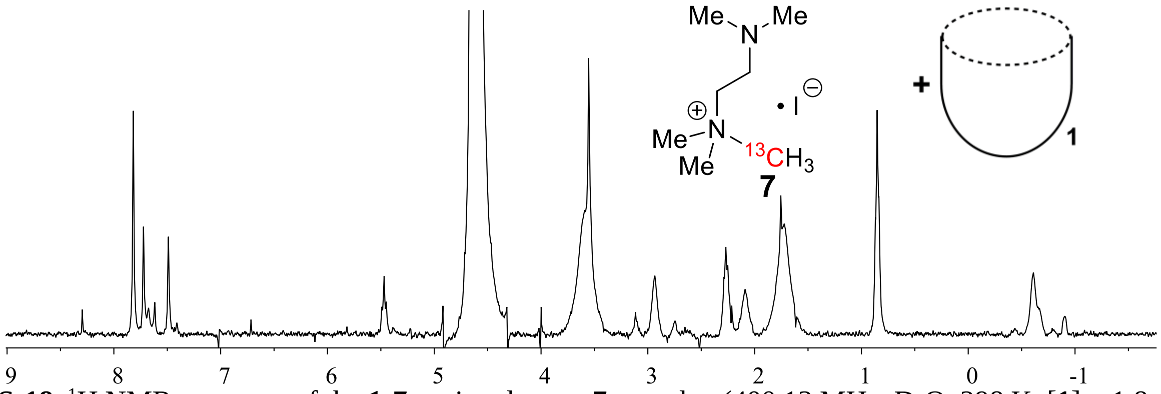

Figure S-18. ^{1}H NMR spectrum of the **1•7** cavitand-guest **7** complex (400.13 MHz, D_2O, 298 K, [**1**] = 1.8 mM, [**7**] = 2.2 mM).

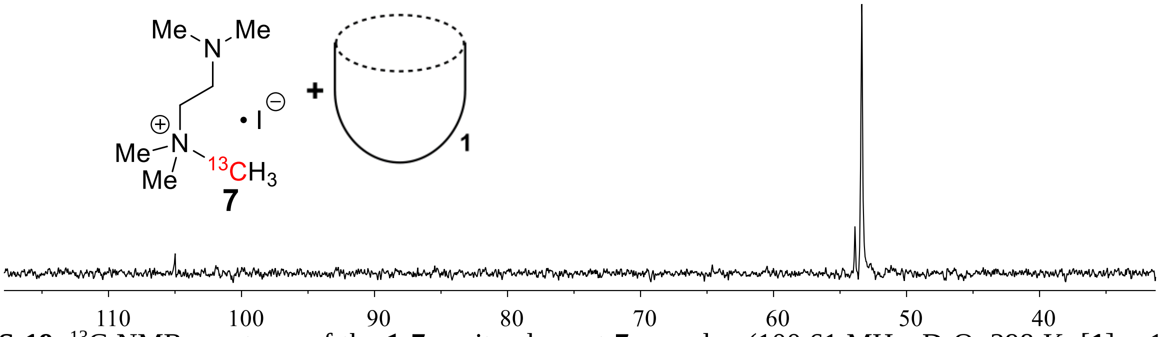

Figure S-19. ^{13}C NMR spectrum of the **1•7** cavitand-guest **7** complex (100.61 MHz, D_2O, 298 K, [**1**] = 1.8 mM, [**7**] = 2.2 mM).

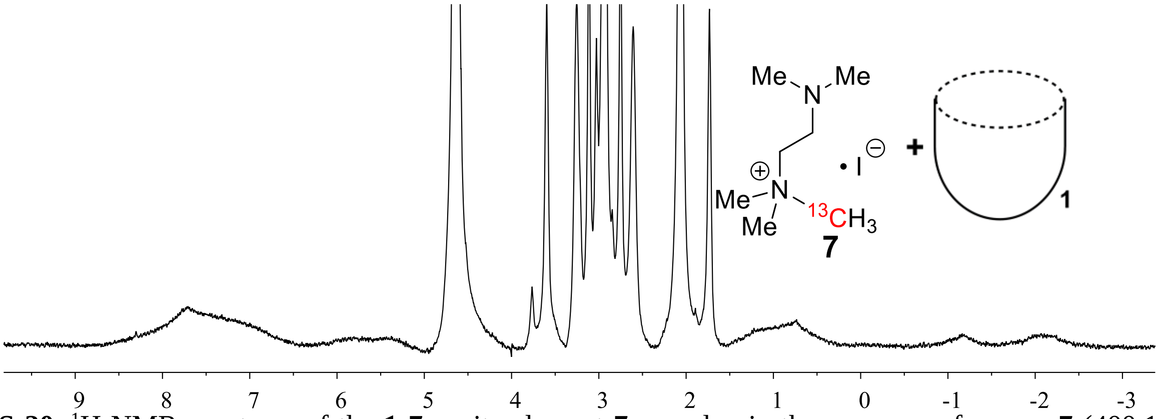

Figure S-20. ^{1}H NMR spectrum of the **1•7** cavitand-guest **7** complex in the presence of excess **7** (400.13 MHz, D_2O, 298 K, [**1**] = 1.8 mM, [**7**] = 10 mM).

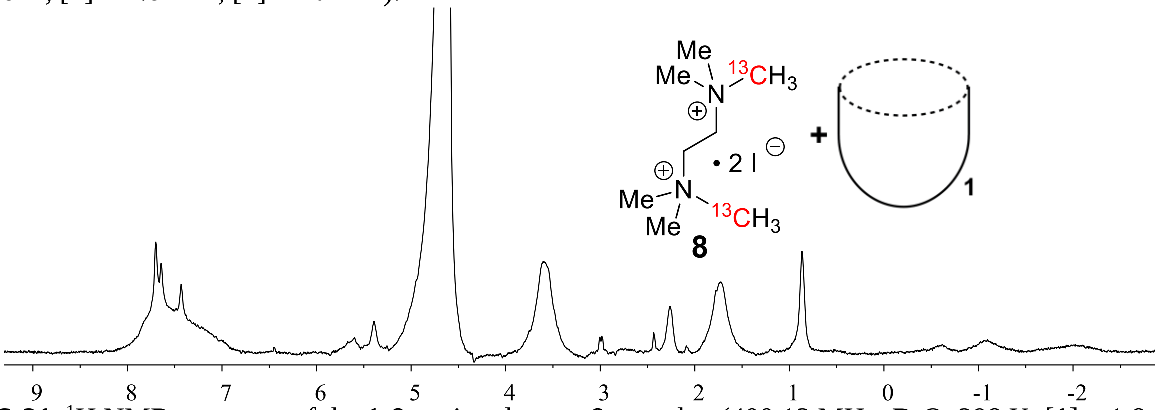

Figure S-21. ^{1}H NMR spectrum of the **1•8** cavitand-guest **8** complex (400.13 MHz, D_2O, 298 K, [**1**] = 1.8 mM, [**8**] = 2.2 mM).

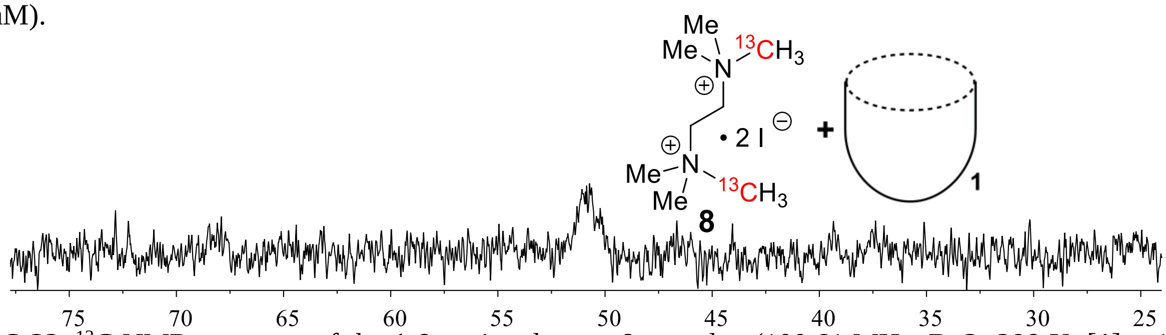

Figure S-22. ^{13}C NMR spectrum of the **1•8** cavitand-guest **8** complex (100.61 MHz, D_2O, 298 K, [**1**] = 1.8 mM, [**8**] = 2.2 mM).

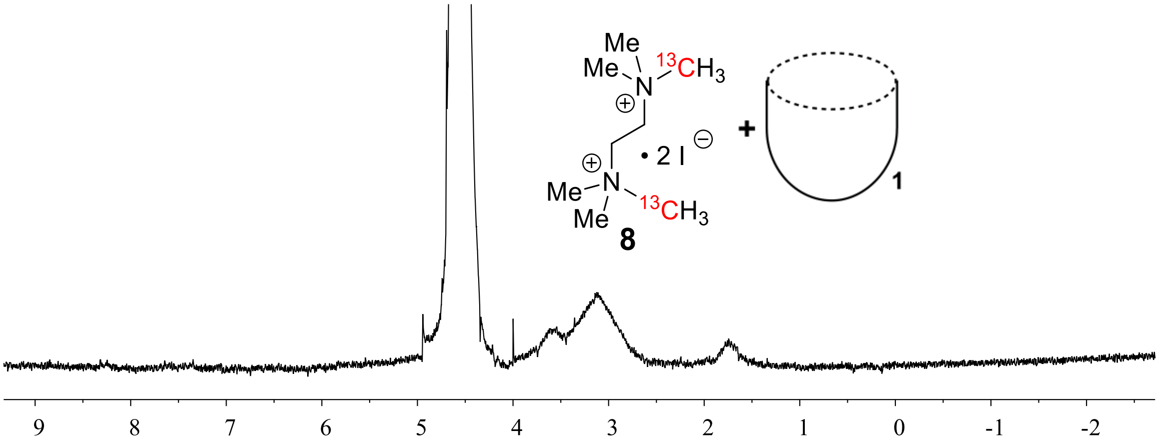

Figure S-23. ^{1}H NMR spectrum of the **1•8** cavitand-guest **8** complex in the presence of excess **8** (400.13 MHz, D_2O, 298 K, [**1**] = 1.8 mM, [**8**] = 5.5 mM).

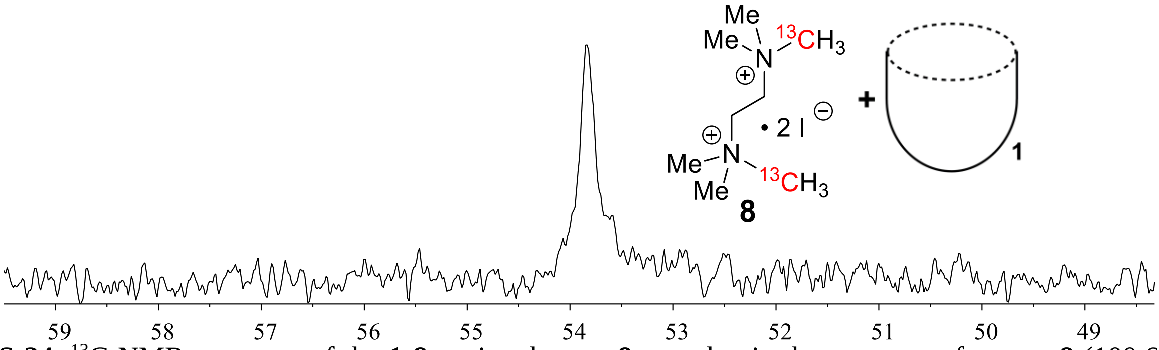

Figure S-24. ^{13}C NMR spectrum of the **1•8** cavitand-guest **8** complex in the presence of excess **8** (100.61 MHz, D_2O, 298 K, [**1**] = 1.8 mM, [**8**] = 5.5 mM).

3. NMR Spectra of Host:Guest Complexes in DMPC/DHPC Lipid Micelles.

Guest 2

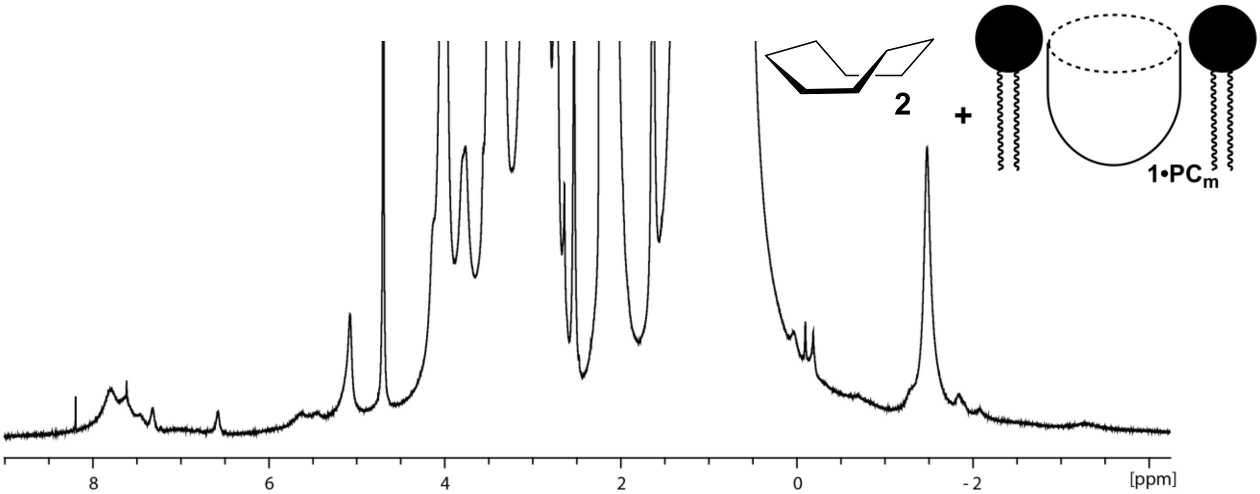

Figure S-25. ^{1}H NMR spectrum of the **1•2** cavitand-cyclooctane complex in DMPC:DHPC lipid micelles (599.88 MHz, 1 mM HEPES/D_2O, 298 K, [**1**] = [**2**] = 1.8 mM, ratio DMPC/DHPC = 3.2:1, 60 mg/mL total lipid concentration).

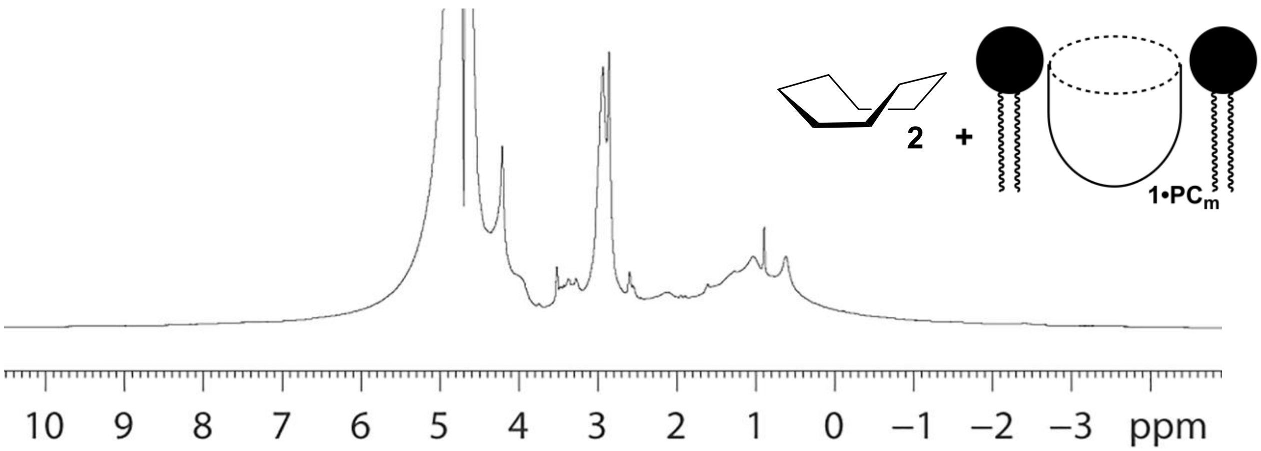

Figure S-26. T2-filtered ^{1}H NMR spectrum of the **1•2** cavitand-cyclooctane complex in DMPC:DHPC lipid micelles (599.88 MHz, 1 mM HEPES/D_2O, 298 K, [**1**] = [**2**] = 1.8 mM, ratio DMPC/DHPC = 3.2:1, 60 mg/mL total lipid concentration).

Guest 3

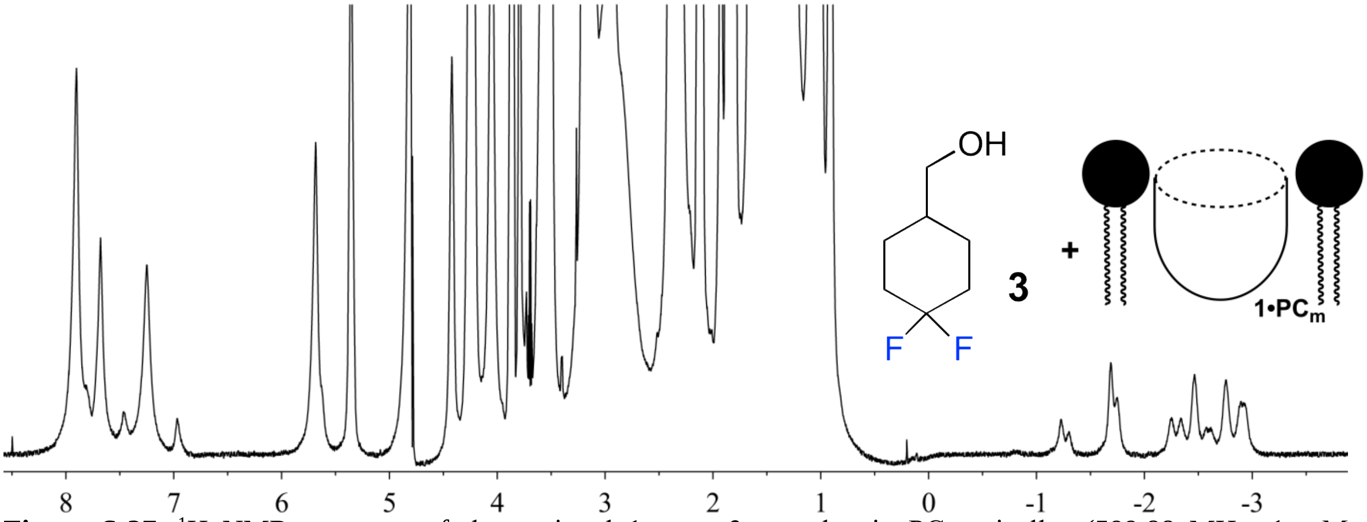

Figure S-27. ^{1}H NMR spectrum of the cavitand **1**•guest **3** complex in PC_m micelles (599.88 MHz, 1 mM HEPES/D_2O, 283 K, [**1**] = 5.8 mM, [**3**] = 39.5 mM, ratio DMPC/DHPC = 3.2:1, 60 mg/mL total lipid concentration).

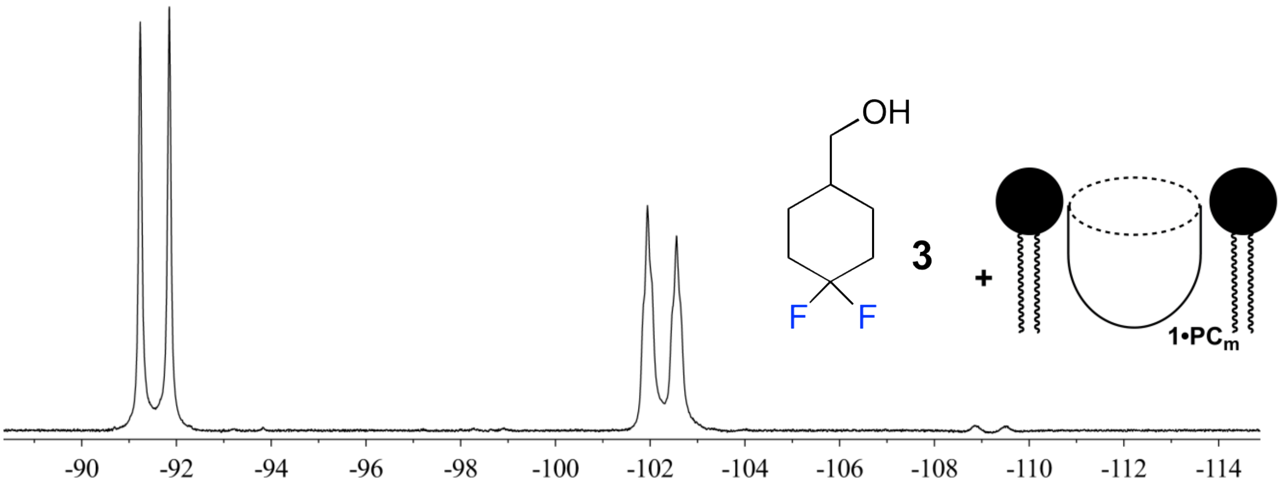

Figure S-28. ^{19}F NMR spectrum of the cavitand **1**•guest **3** complex in PC_m micelles (1 mM HEPES/D_2O, 376.50 MHz, 298 K, [**1**] = 5.8 mM, [**3**] = 39.5 mM, ratio DMPC/DHPC = 3.2:1, 60 mg/mL total lipid concentration).

Guest 4

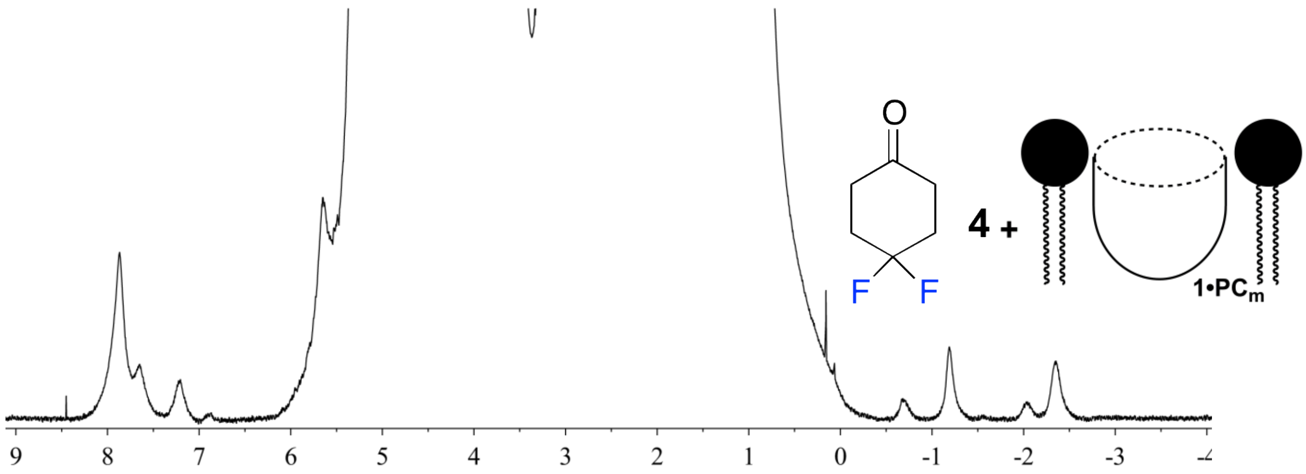

Figure S-29. ^{1}H NMR spectrum of the cavitand **1**•guest **4** complex in PC$_m$ micelles (599.88 MHz, 1 mM HEPES/D_2O, 283 K, [**1**] = 5.8 mM, [**4**] = 39.5 mM, ratio DMPC/DHPC = 3.2:1, 60 mg/mL total lipid concentration).

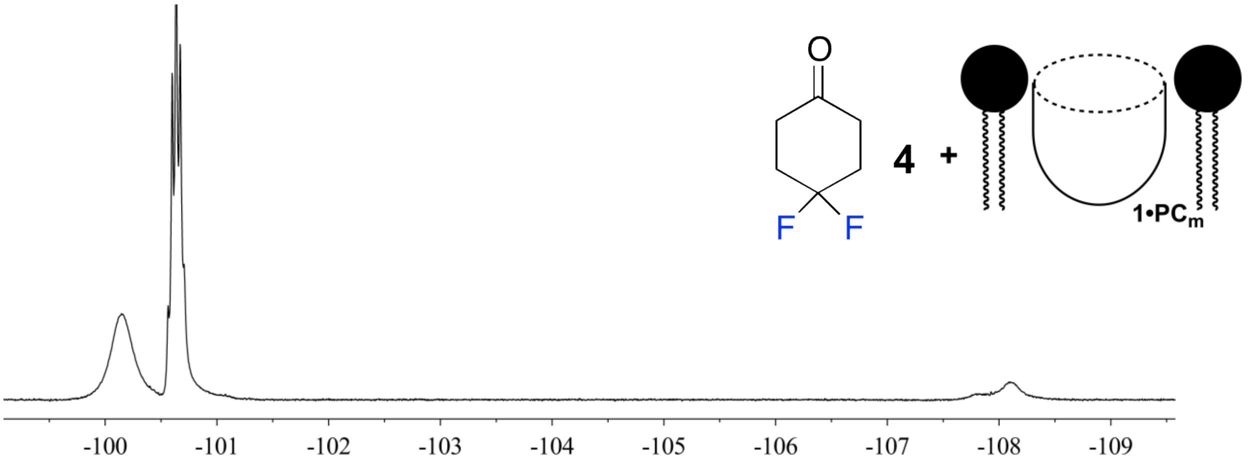

Figure S-30. ^{19}F NMR spectrum of the cavitand **1**•guest **4** complex in PC$_m$ micelles (1 mM HEPES/D_2O, 376.50 MHz, 298 K, [**1**] = 5.8 mM, [**4**] = 39.5 mM, ratio DMPC/DHPC = 3.2:1, 60 mg/mL total lipid concentration);

Guest 6

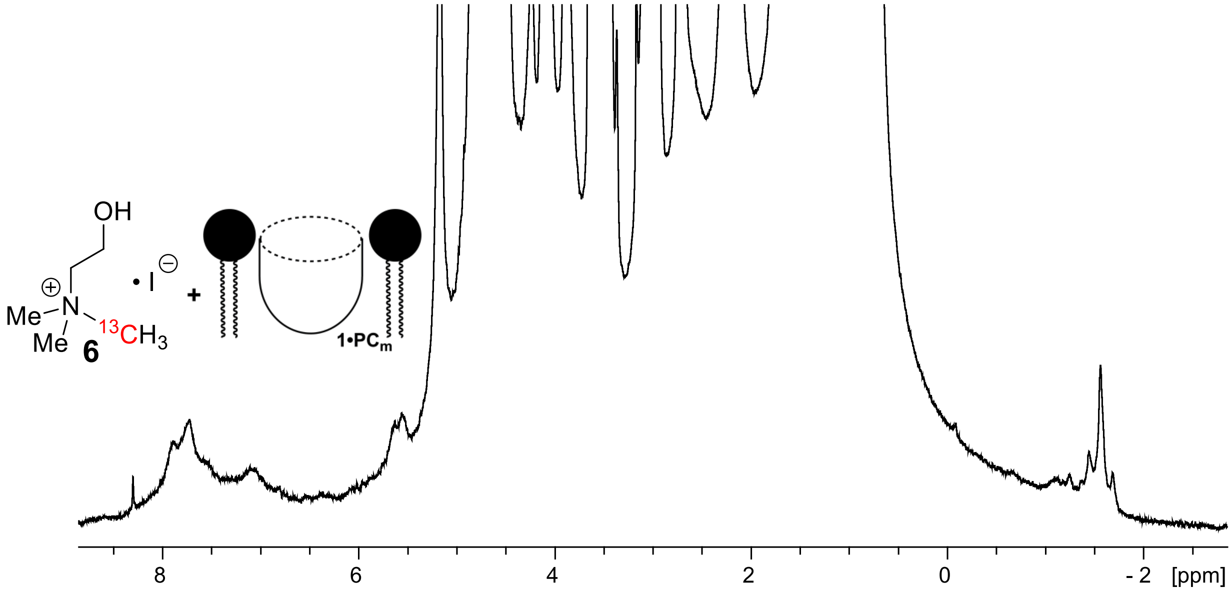

Figure S-31. ^{1}H NMR spectrum of the **1•6** cavitand-choline **6** complex in DMPC:DHPC lipid micelles (599.88 MHz, 1 mM HEPES/D_2O, 283 K, [**1**] = 5.8 mM, [**6**] = 16.0 mM, ratio DMPC/DHPC = 3.2:1, 60 mg/mL total lipid concentration).

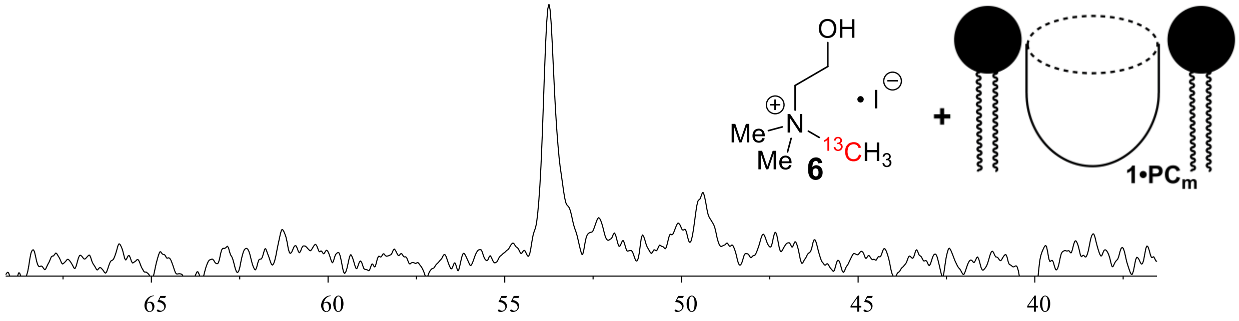

Figure S-32. ^{13}C NMR spectrum of the **1•6** cavitand-choline **6** complex in DMPC:DHPC lipid micelles (100.61 MHz, 1 mM HEPES/D_2O, 283 K, [**1**] = 5.8 mM, [**6**] = 16.0 mM, ratio DMPC/DHPC = 3.2:1, 60 mg/mL total lipid concentration).

Guest 7

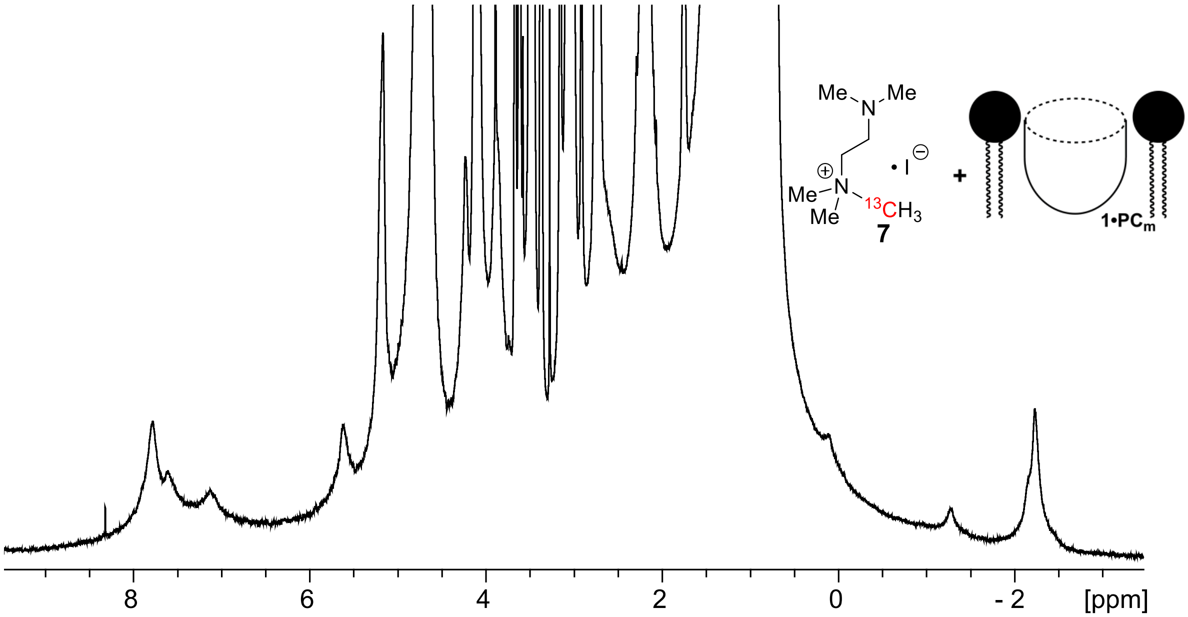

Figure S-33. ^{1}H NMR spectrum of the **1•7** cavitand-guest **7** complex in DMPC:DHPC lipid micelles (599.88 MHz, 1 mM HEPES/D_2O, 283 K, [**1**] = 5.8 mM, [**7**] = 16 mM, ratio DMPC/DHPC = 3.2:1, 60 mg/mL total lipid concentration).

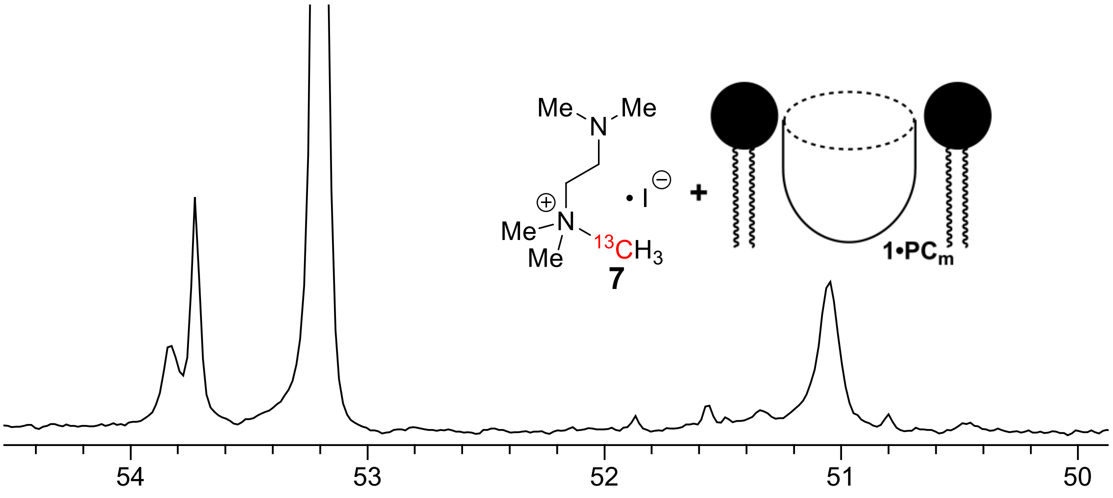

Figure S-34. ^{13}C NMR spectrum of the **1•7** cavitand-guest **7** complex in DMPC:DHPC lipid micelles (100.61 MHz, 1 mM HEPES/D_2O, 283 K, [**1**] = 5.8 mM, [**7**] = 16 mM, ratio DMPC/DHPC = 3.2:1, 60 mg/mL total lipid concentration).

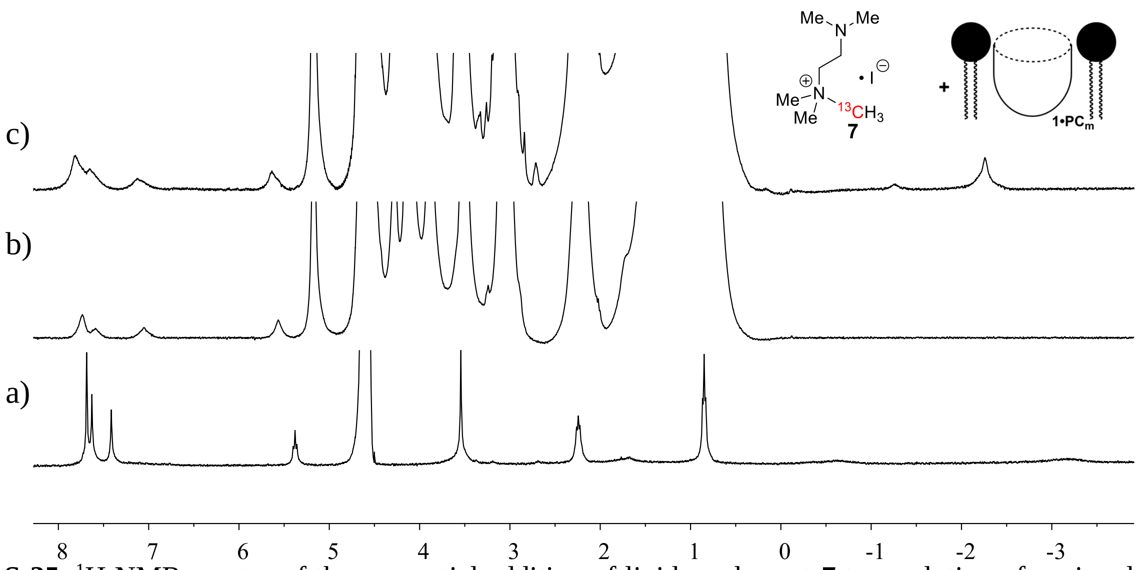

Figure S-35. ^{1}H NMR spectra of the sequential addition of lipids and guest **7** to a solution of cavitand **1** (400.13 MHz, 1 mM HEPES/D_2O, 298 K). a) 1.8 mM of cavitand **1**; b) 1.8 mM of cavitand **1** + DMPC/DHPC lipids, ratio DMPC/DHPC = 3.2:1, 60 mg/mL total lipid concentration; c) 1.8 mM of cavitand **1** + DMPC/DHPC lipids =2.2 mM guest **7**.

Guest 8

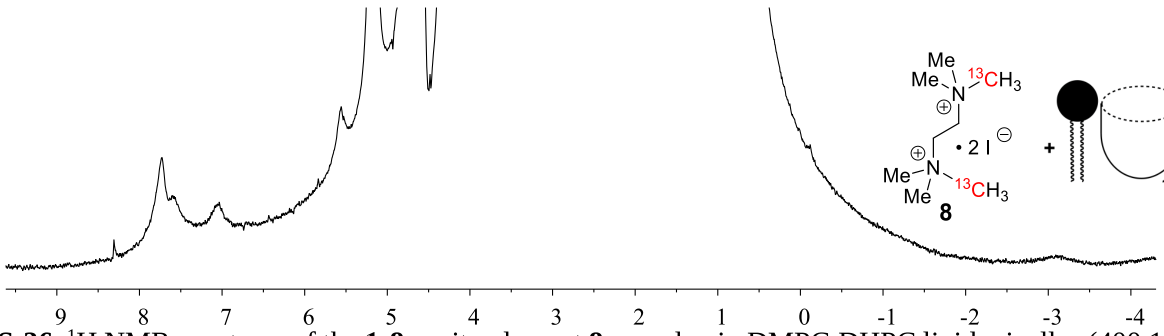

Figure S-36. ^{1}H NMR spectrum of the **1•8** cavitand-guest **8** complex in DMPC:DHPC lipid micelles (400.13 MHz, 1 mM HEPES/D_2O, 298 K, [**1**] = 1.8 mM, [**8**] = 5.4 mM, ratio DMPC/DHPC = 3.2:1, 60 mg/mL total lipid concentration).

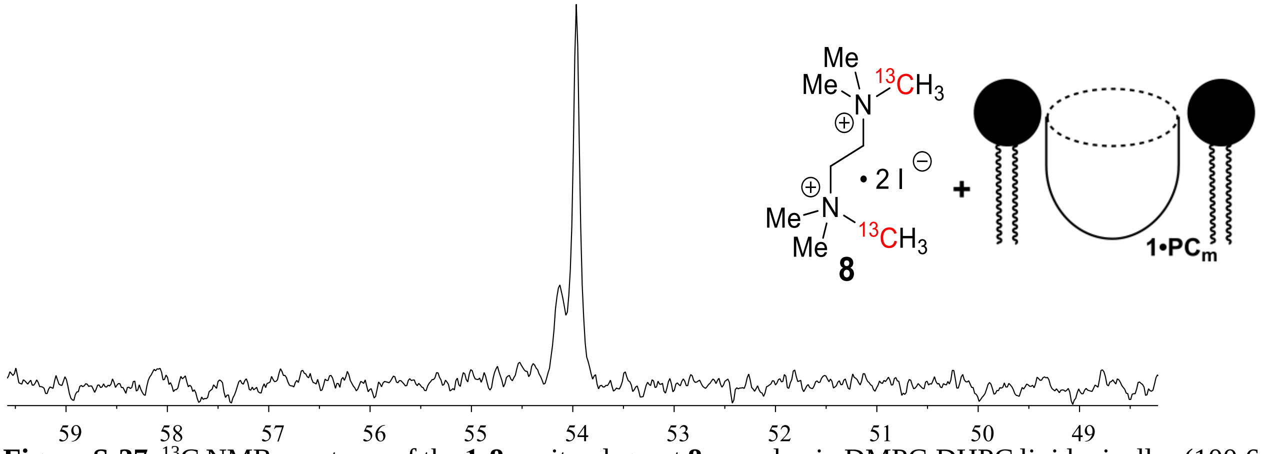

Figure S-37. ^{13}C NMR spectrum of the **1•8** cavitand-guest **8** complex in DMPC:DHPC lipid micelles (100.61 MHz, 1 mM HEPES/D_2O, 298 K, [**1**] = 1.8 mM, [**8**] = 5.4 mM, ratio DMPC/DHPC = 3.2:1, 60 mg/mL total lipid concentration).

4. Fitting model for EXSY Experiments

A standard two-site exchange model is used to analyze the EXSY data. In this model, the guest can exchange between a free and bound state, A and B, respectively:

$$A \underset{k_b}{\overset{k_f}{\rightleftharpoons}} B$$

k_b is the exchange constant from B to A, while k_f is the exchange constant for A to B. At equilibrium, the forward and reverse exchange rates are equal. Therefore $k_b[B] = k_f[A]$, or in terms of the equilibrium constant:

$$\frac{[B]}{[A]} = \frac{k_f}{k_b} = K_{eq} ,$$

During the mixing time of the EXSY experiment, the concentration of A can change in three ways: first, A can become bound, decreasing its contribution to the signal intensity over the mixing time; second, A can relax due to T_1, also decreasing its intensity; third, a bound ligand B can be released, becoming A and adding to its signal intensity. This can be described by the following first-order differential equation:

$$\frac{d[A]}{dt} = -k_f[A] - \frac{[A]}{T_{1A}} + k_b[B]$$

The signal from B varies in a similar way, giving

$$\frac{d[B]}{dt} = -k_b[B] - \frac{[B]}{T_{1B}} + k_f[A]$$

Each exchange spectrum contains two diagonal peaks, AA and BB, and two crosspeaks, AB and BA; the first letter refers to the frequency in f_1 of the EXSY spectrum, while the second gives the frequency in f_2 (hence AB would be the cross peak at $\{\nu_A, \nu_B\}$). The above set of coupled, first-order, linear differential equations can be solved using standard methods subject to the following initial conditions at the start of the EXSY mixing time:

AA and AB: $[A(t=0)] = A_0$, $[B(t=0)] = 0$

BB and BA: $[A(t=0)] = 0$, $[B(t=0)] = B_0$

giving the following expressions for the diagonal and cross peak intensities in terms of the EXSY mixing time (t):

$$AA(t) = A_0 e^{-t/T_1} \frac{k_b + e^{-(k_b+k_f)t} k_f}{k_b + k_f} \overset{\text{short time}}{\approx} A_0\left(1 - \tfrac{t}{T_1} - k_f t\right)$$

$$AB(t) = A_0 e^{-t/T_1} \frac{\left(1 - e^{-(k_b+k_f)t}\right) k_f}{k_b + k_f} \overset{\text{short time}}{\approx} A_0 k_f t$$

$$BB(t) = B_0 e^{-t/T_1} \frac{e^{-(k_b+k_f)t} k_b + k_f}{k_b + k_f} \overset{\text{short time}}{\approx} B_0\left(1 - \tfrac{t}{T_1} - k_b t\right)$$

$$BA(t) = B_0 e^{-t/T_1} \frac{\left(1 - e^{-(k_b+k_f)t}\right) k_b}{k_b + k_f} \overset{\text{short time}}{\approx} B_0 k_b t$$

where it has been assumed that $T_{1A} = T_{1B}$. The initial rate (short time) solutions are shown to the right. By extracting one-dimensional slices showing the greatest peak intensity from 2D spectra recorded with different mixing times, it is possible to fit the above set of equations and determine both k_b and k_f.

5. 2D Exchange NMR Spectra in Water and in Lipid Micelles

Guest 3

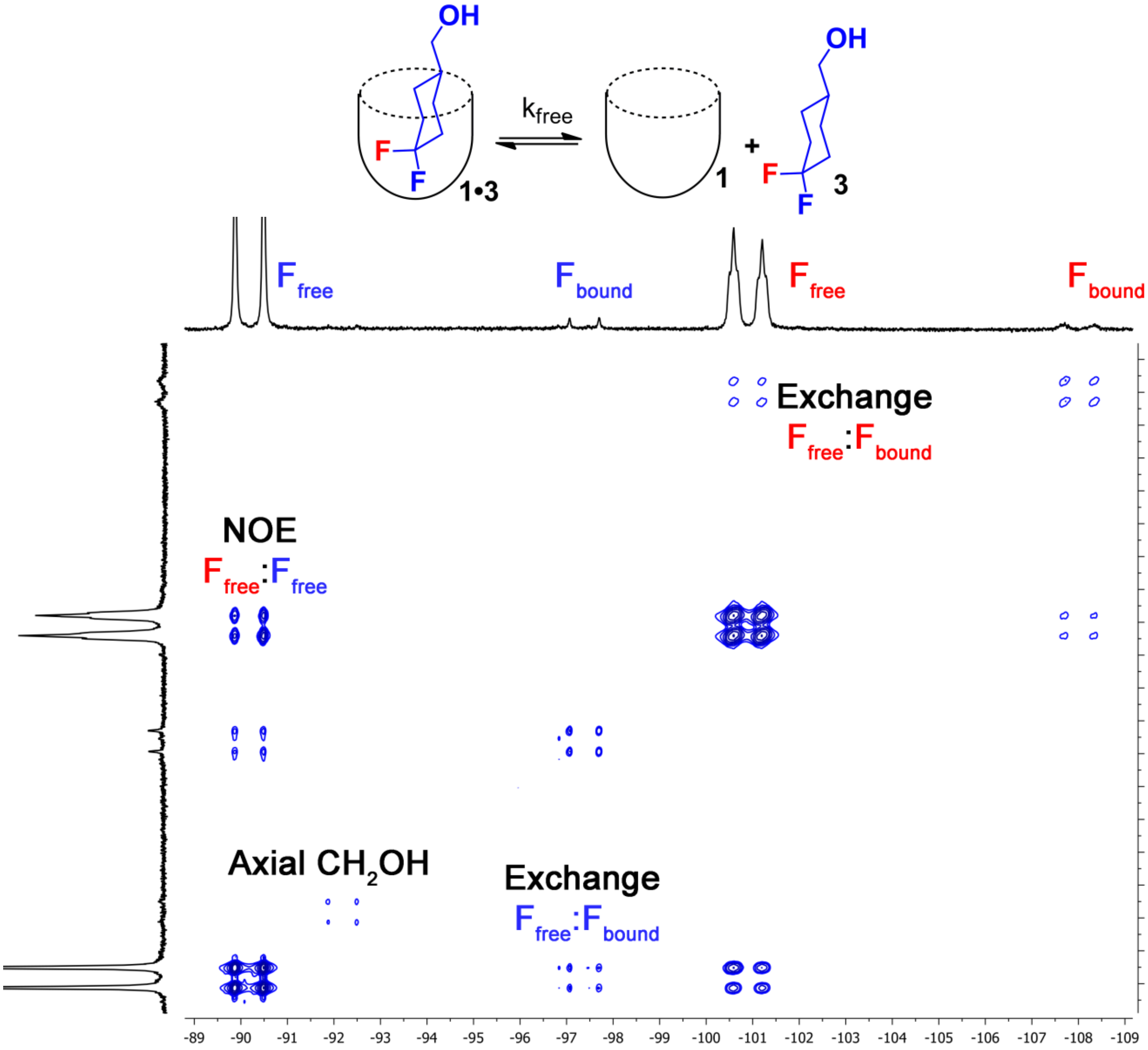

Figure S-38. Full ^{19}F EXSY spectrum of the cavitand **1**•guest **3** complex in pure D_2O with peak assignments (D_2O, 150.84 MHz, 298 K, mixing time = 150 ms, [**1**] = 5.8 mM, [**3**] = 39.5 mM)

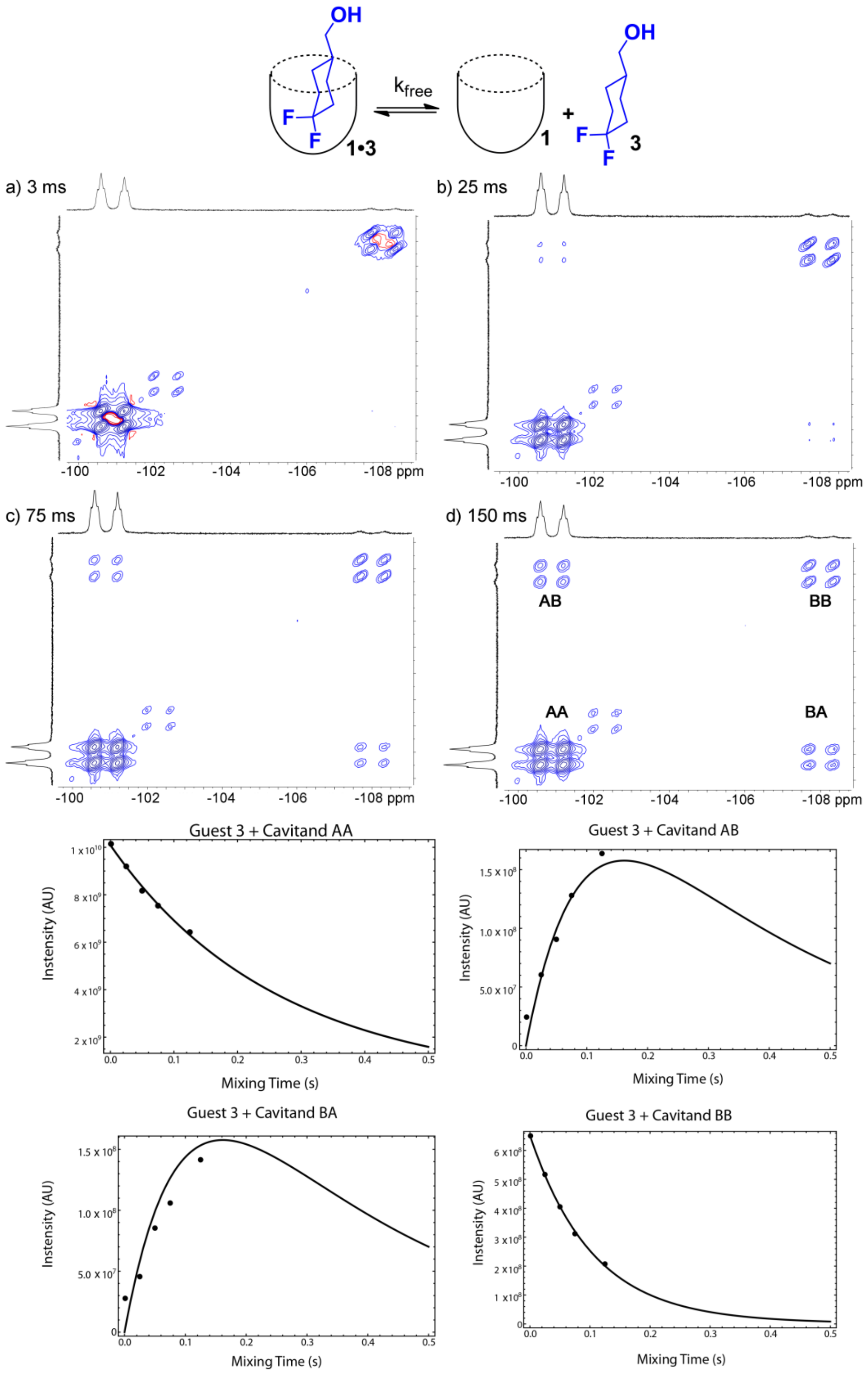

Figure S-39. Representative ^{19}F EXSY spectra of the cavitand **1**•guest **3** complex in pure D_2O with varying mixing times (D_2O, 376.50 MHz, 298 K, [**1**] = 5.8 mM, [**3**] = 39.5 mM); fitted plots of peak intensity correlated with mixing time for each diagonal and crosspeak used to calculate the exchange rate.

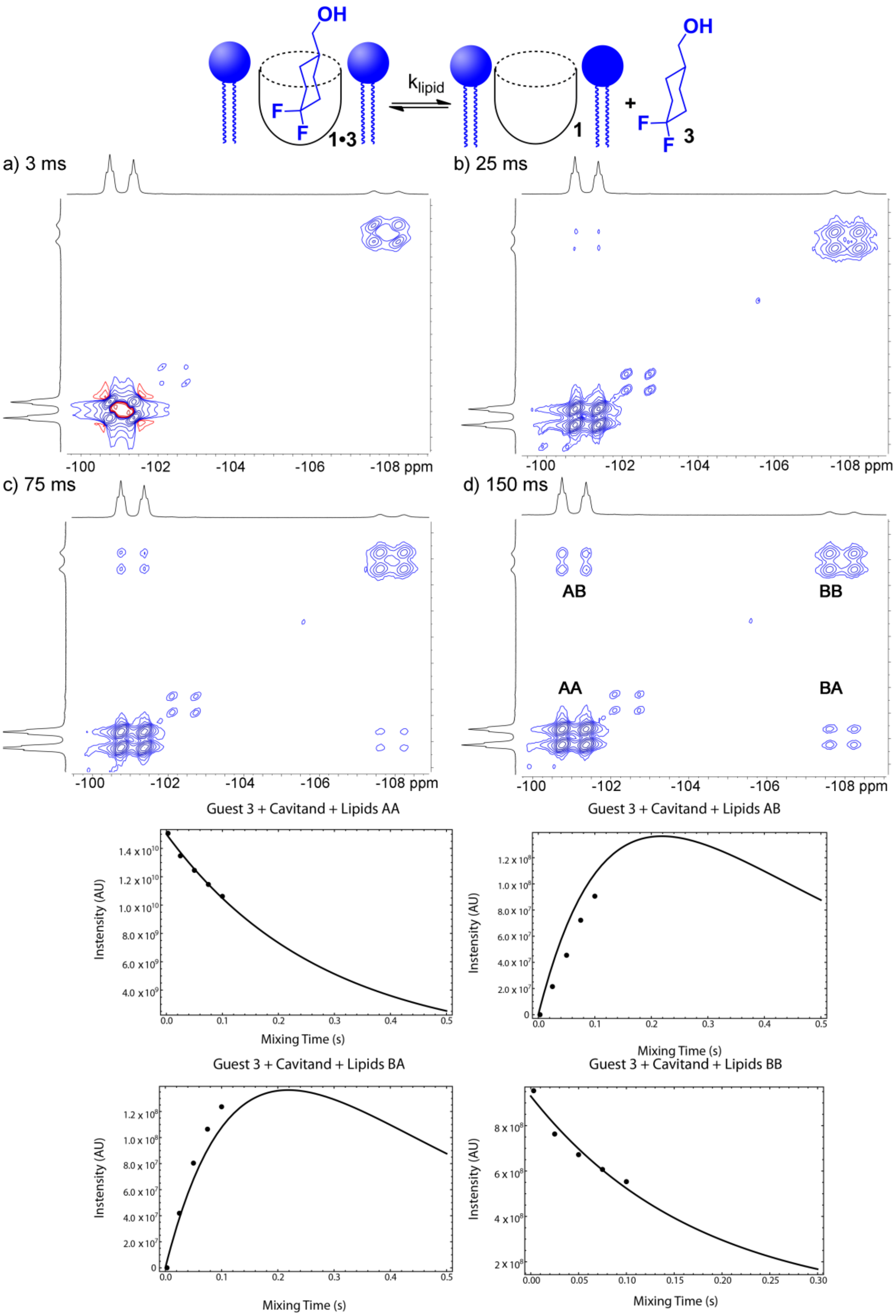

Figure S-40. Representative ^{19}F EXSY spectra of the cavitand **1**•guest **3** complex in the PC_m micelles with varying mixing times **(**1 mM HEPES/D_2O, 376.50 MHz, 298 K, [**1**] = 5.8 mM, [**3**] = 39.5 mM, ratio DMPC/DHPC = 3.2:1, 60 mg/mL total lipid concentration); fitted plots of peak intensity correlated with mixing time for each diagonal and crosspeak used to calculate the exchange rate.

Guest 4

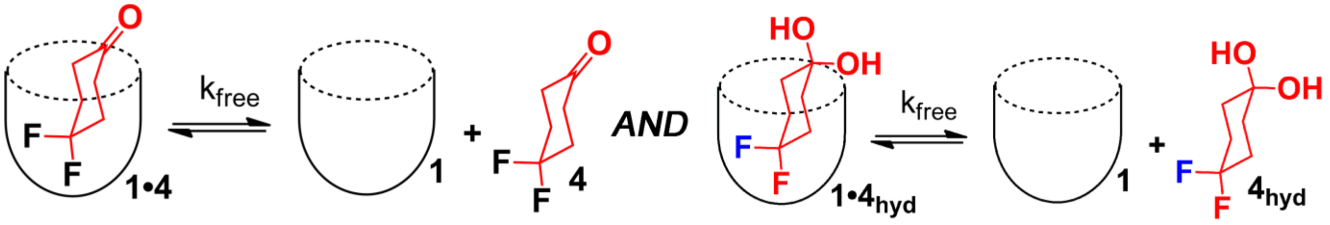

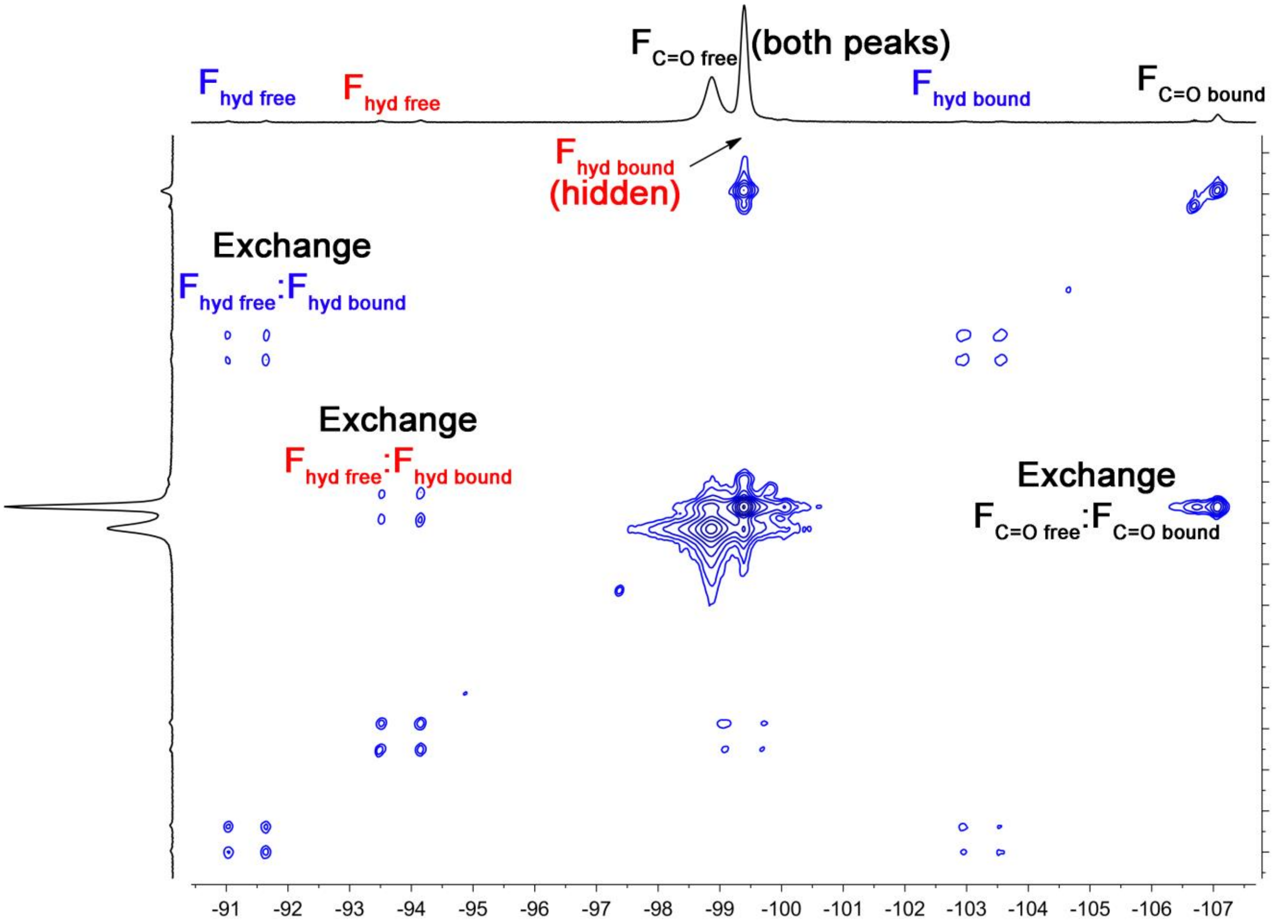

Figure S-41. Full ^{19}F EXSY spectrum of the cavitand **1**•guest **4** complex in pure D_2O with peak assignments (D_2O, 150.84 MHz, 298 K, mixing time = 150 ms, [**1**] = 5.8 mM, [**4**] = 39.5 mM).

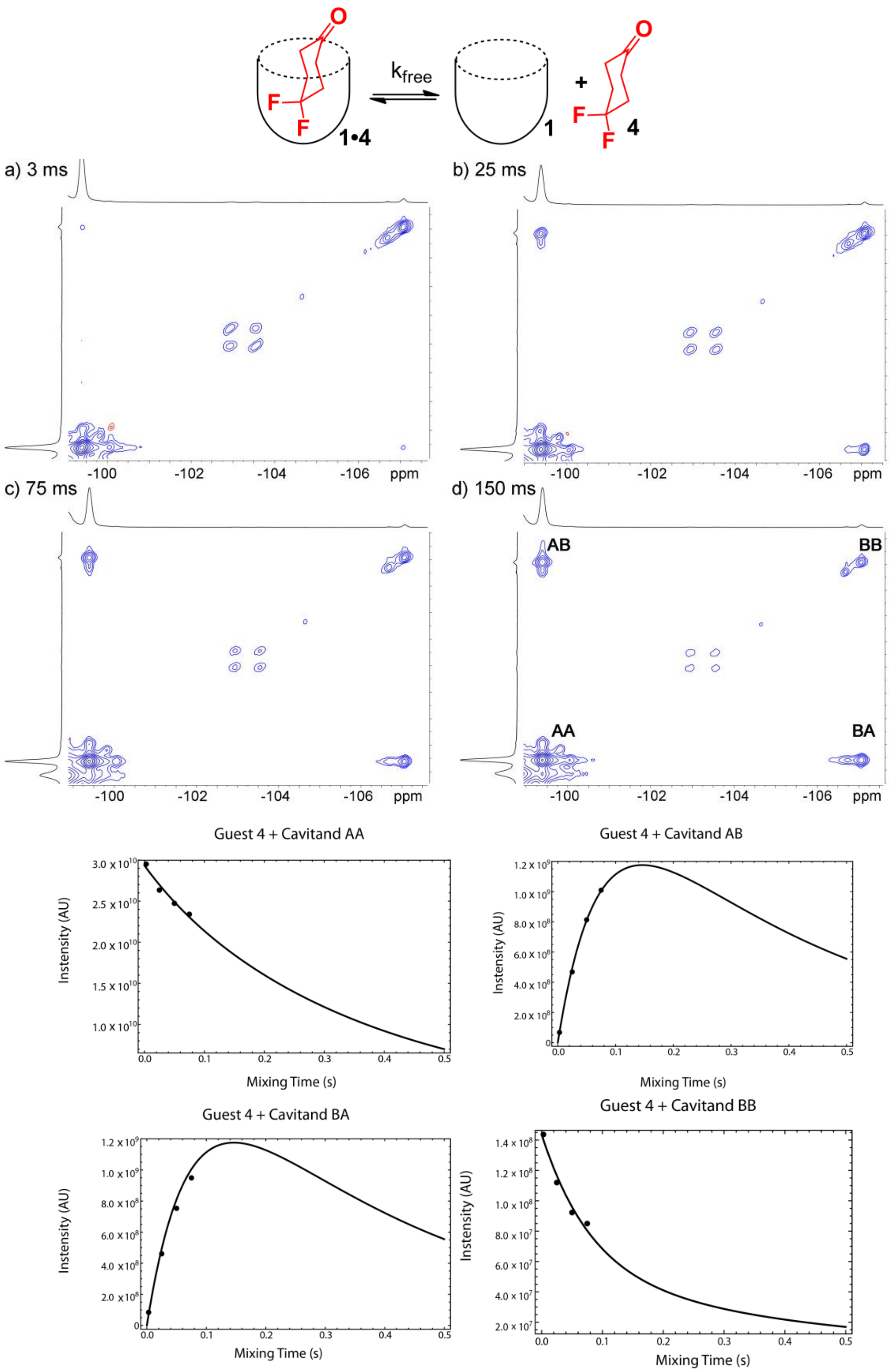

Figure S-42. Representative ^{19}F EXSY spectra of the cavitand **1**•guest **4** complex in pure D_2O with varying mixing times (D_2O, 376.50 MHz, 298 K, [**1**] = 5.8 mM, [**4**] = 39.5 mM); fitted plots of peak intensity correlated with mixing time for each diagonal and crosspeak used to calculate the exchange rate.

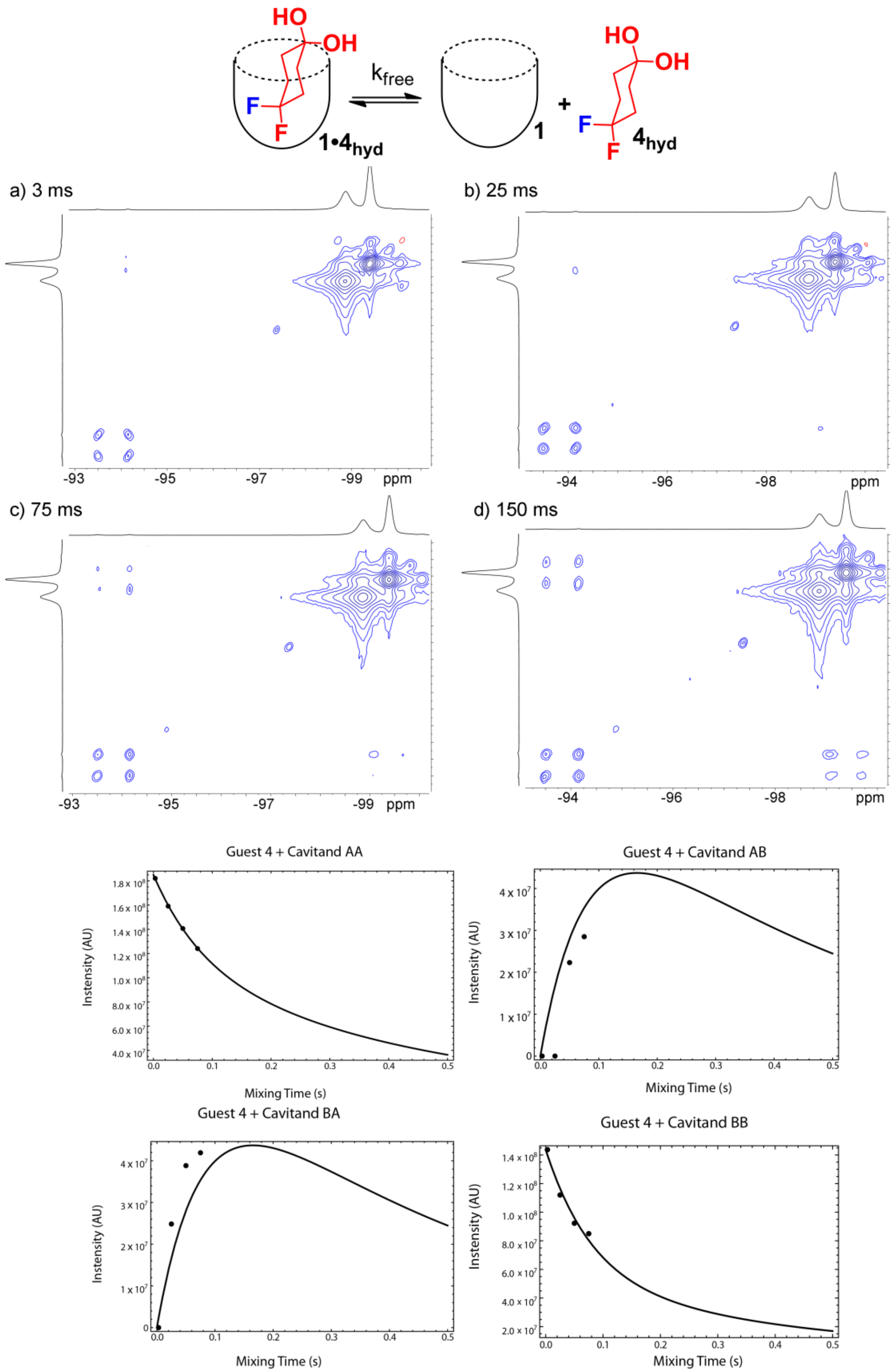

Figure S-43. Representative ^{19}F EXSY spectra of the cavitand **1**•guest **4** complex in pure D_2O with varying mixing times (D_2O, 376.50 MHz, 298 K, [**1**] = 5.8 mM, [**4**] = 39.5 mM) illustrating the exchange peaks of the hydrate; fitted plots of peak intensity correlated with mixing time for each diagonal and crosspeak used to calculate the exchange rate.

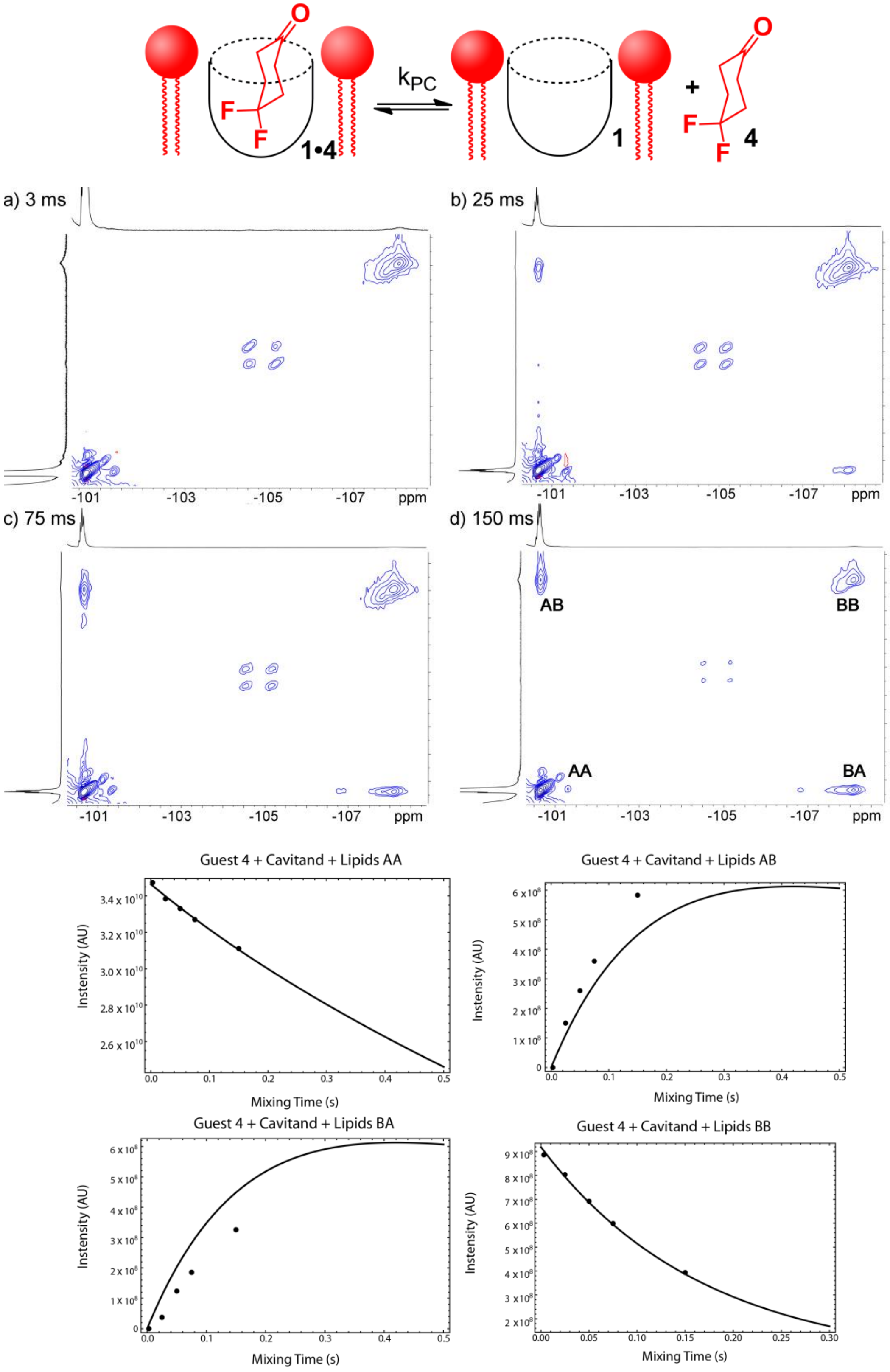

Figure S-44. Representative ^{19}F EXSY spectra of the cavitand **1**•guest **4** complex in the PC_m micelles with varying mixing times (1 mM HEPES/D_2O, 376.50 MHz, 298 K, [**1**] = 5.8 mM, [**4**] = 39.5 mM, ratio DMPC/DHPC = 3.2:1, 60 mg/mL total lipid concentration); fitted plots of peak intensity correlated with mixing time for each diagonal and crosspeak used to calculate the exchange rate.

Guest 6

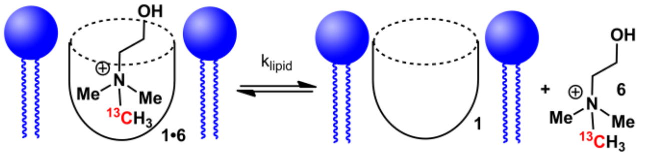

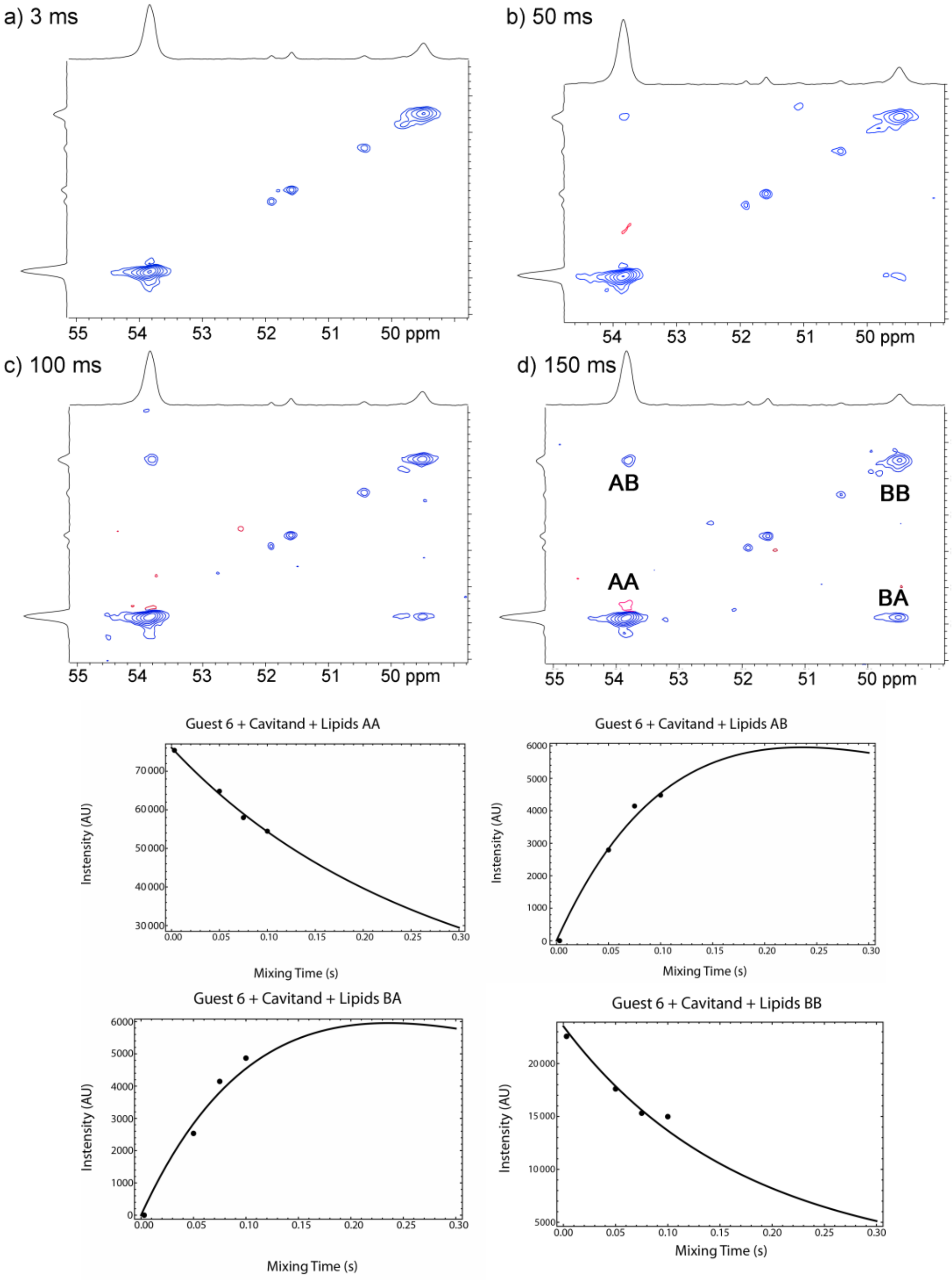

Figure S-45. Representative ^{13}C EXSY spectra of the cavitand **1**•guest **6** complex in the PC$_m$ micelles with varying mixing times (1 mM HEPES/D_2O, 150.84 MHz, 298 K, [**1**] = 5.8 mM, [**6**] = 16 mM, ratio DMPC/DHPC = 3.2:1, 60 mg/mL total lipid concentration); fitted plots of peak intensity correlated with mixing time for each diagonal and crosspeak used to calculate the exchange rate.

Guest 7

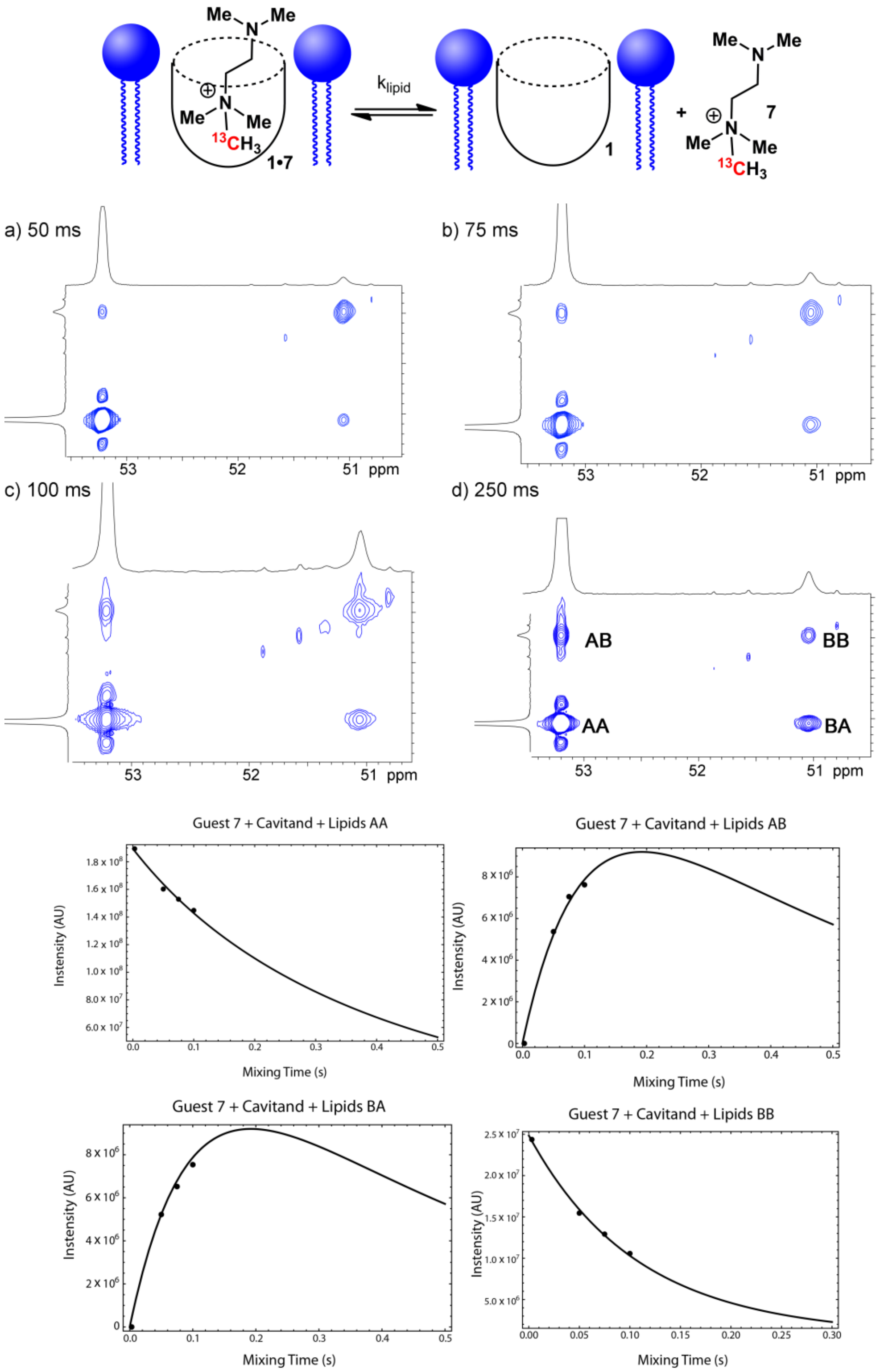

Figure S-46. Representative ^{13}C EXSY spectra of the cavitand **1**•guest **7** complex in the PC$_m$ micelles with varying mixing times (1 mM HEPES/D_2O, 150.84 MHz, 298 K, [**1**] = 5.8 mM, [**7**] = 16 mM, ratio DMPC/DHPC = 3.2:1, 60 mg/mL total lipid concentration); fitted plots of peak intensity correlated with mixing time for each diagonal and crosspeak used to calculate the exchange rate.

6. NMR Spectra in Magnetically Ordered DMPC/DHPC Bicelles

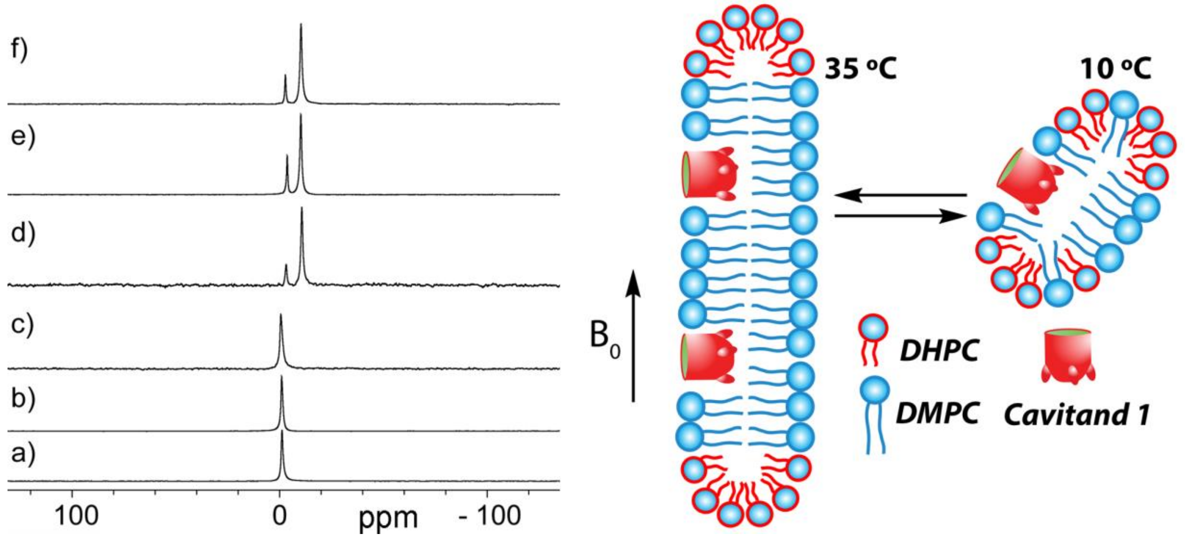

Figure S-47. Temperature dependence of the lipid aggregate. ^{31}P NMR spectra of the DMPC/DHPC aggregates a) alone, 283 K; b) + 5 mM **1**, 283 K; c) + 5 mM **1** + 7 mM guest **5**, 283 K; d) alone, 303 K; e) + 5 mM 1, 303 K; f) + 5 mM **1** + 7 mM guest **5**, 303 K. Ratio DMPC/DHPC = 3.2:1, 150 mg/mL total lipid concentration, 162.07 MHz, 2.5 mM HEPES/D_2O.

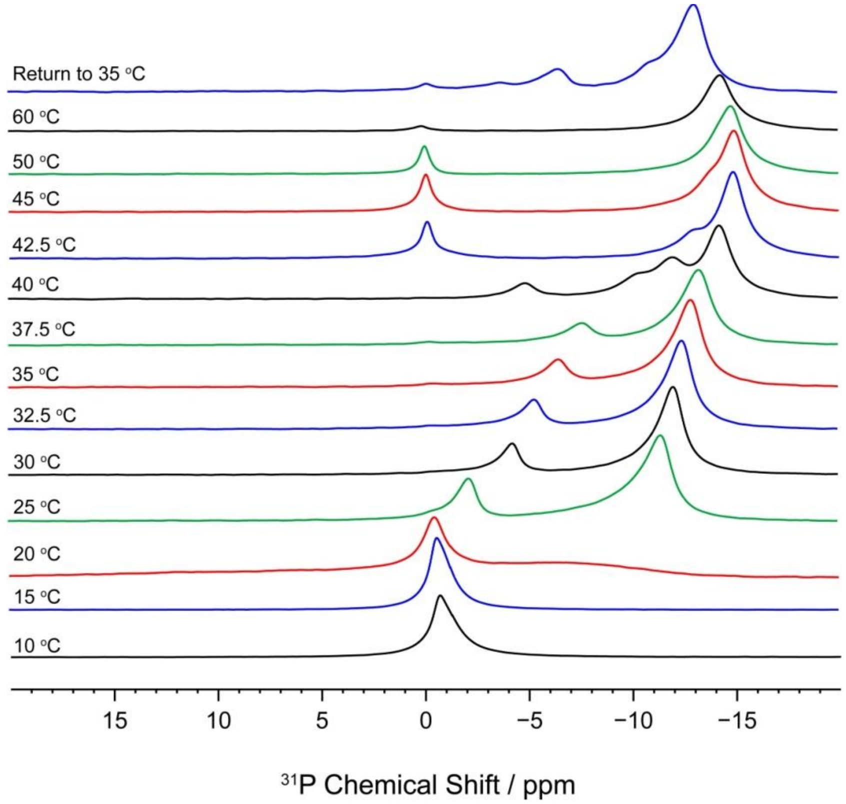

Figure S-48. Temperature-dependent ^{31}P spectra of DMPC/DHPC bicelles. The two peaks indicative of bicelle formation display maximum splitting at 35 °C, the temperature at which all future experiments were performed, unless otherwise indicated. Low temperatures display a single peak at 0 ppm, indicative of micelle formation. Ratio DMPC/DHPC = 3.2:1, 150 mg/mL total lipid concentration, 162.07 MHz, 2.5 mM HEPES/D_2O.

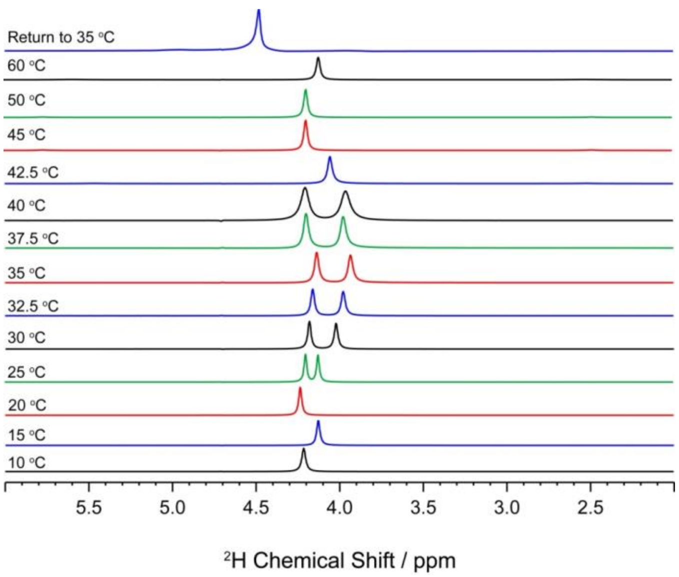

Figure S-49. Temperature-dependent ^{2}H spectra of DMPC/DHPC bicelles. The two peaks indicative of bicelle formation display maximum splitting at 35 °C, the temperature at which all future experiments were performed, unless otherwise indicated. Low temperatures display a single resonance, indicative of micelle formation. Ratio DMPC/DHPC = 3.2:1, 150 mg/mL total lipid concentration, 92.09 MHz, 2.5 mM HEPES/D_2O.

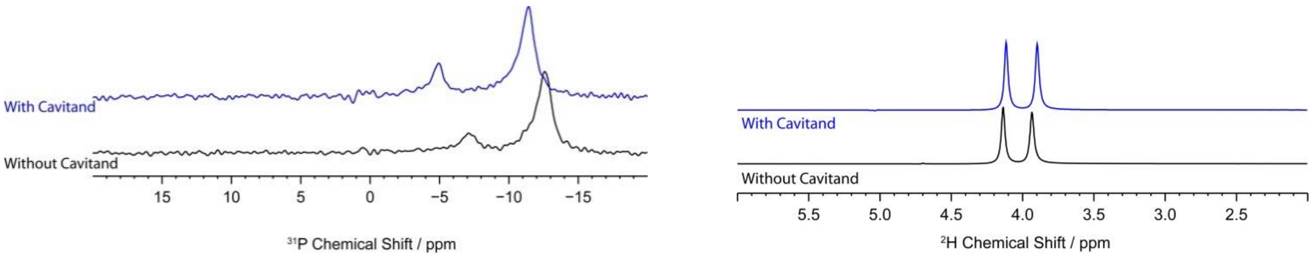

Figure S-50. ^{31}P and ^{2}H spectra of bicelles with and without cavitand added. Ratio DMPC/DHPC = 3.2:1, 150 mg/mL total lipid concentration, 162.07/ 92.09 MHz, 2.5 mM HEPES/D_2O.

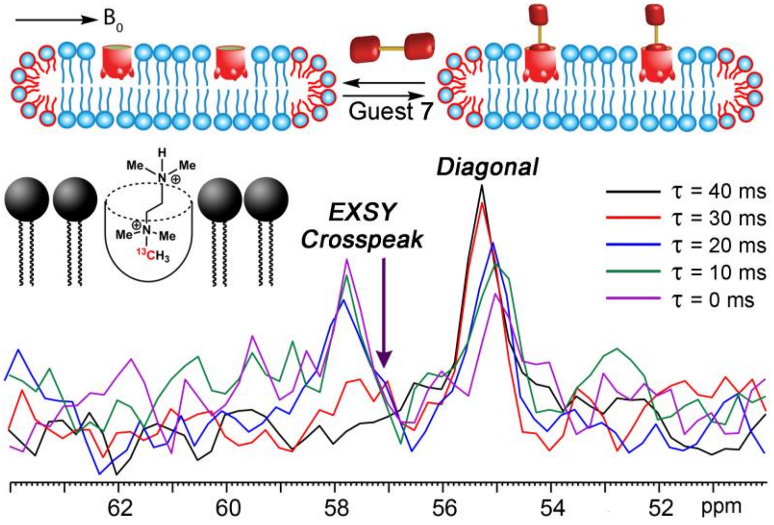

Figure S-51. In/out exchange of guest **7** in the magnetically ordered bicelle system PC_b. Extracted 1D slices of the 2D ^{13}C-^{13}C EXSY NMR spectra at mixing time τ = 0 ms - τ = 40 ms of **1•7•PC$_b$**; (2.5 mM HEPES/D_2O, 100.69 MHz, 298 K, [**1**] = 20 mM, [**7**] = 36 mM, ratio DMPC/DHPC = 3.2:1, 150 mg/mL total lipid concentration). Change in diagonal and crosspeak intensity shown. Slices extracted from F2, δ = 55 ppm.
